# Coordinated single-nucleus responses for quantitative disease resistance involve a calcium-associated switch in transcriptional noise

**DOI:** 10.1186/s13059-025-03906-x

**Published:** 2025-12-31

**Authors:** Darcy A. B. Jones, Florent Delplace, Mehdi Khafif, Matilda Zaffuto, Tou Cheu Xiong, Adelin Barbacci, Sylvain Raffaele

**Affiliations:** 1 LIPME, INRAE - CNRS, Université de Toulouse, Castanet Tolosan, France; 2 MIAT, INRAE, Université de Toulouse, Castanet Tolosan, France; 3 IPSIM, Univ Montpellier, CNRS, INRAE, Institut Agro, Montpellier, France

**Keywords:** Plant immunity, Fungal pathogen, Single-cell, Transcriptional noise, Calcium signaling

## Abstract

**Background:**

Efficient plant immune response requires concerted reprogramming of cellular transcriptomes both globally and locally at the site of infection. Upon inoculation by the fungal pathogen *Sclerotinia sclerotiorum*, plants show quantitative disease resistance characterized by transcriptional reprogramming of numerous genes with small phenotypic effect.

**Results:**

To study transcriptional heterogeneity across cells during quantitative disease resistance, we combine end-point single-nucleus RNA-sequencing and time-course RNA-seq of mock-treated and *S. sclerotiorum-*inoculated *Arabidopsis thaliana* leaves. We observe heterogeneity of plant immune responses across cell types and in sub-populations of mesophyll cells, and reconstruct the sequence of immune responses activation over time. The quantification of gene expression heterogeneity reveals a transient increase in intrinsic transcriptional noise followed by the activation of key defense genes and the rise of extrinsic transcriptional noise in infected cells. Using the R-GECO1 cytoplasmic calcium reporter, we find that the intensity of calcium variations upon *S. sclerotiorum* inoculation coincides with variations to transcriptional noise in space and time.

**Conclusions:**

These results provide evidence that stochastic cell–cell variability plays a key role in commitment to plant immunity and in the coordination of plant defense at the organ scale. Our study offers new insights into the mechanisms underlying plasticity and robustness of plant immune responses that can inform the design of strategies to reduce pathogen damage to crops in unstable environments.

**Supplementary Information:**

The online version contains supplementary material available at 10.1186/s13059-025-03906-x.

## Background

Plant tissues interacting with pathogenic microorganisms are inherently heterogeneous, composed of diverse cell types with distinct capacities to respond [1]. For instance, the colonization of plant tissues by necrotrophic pathogens actively killing host cells releases cell wall tension locally, causing fluctuations of tensile stress in walls of healthy cells distant from the infection site. This propagation of mechanical cues potentiates immunity in healthy cells and contributes to the spatial organization of plant defense responses [2]. During host colonization, colonies of the fungal pathogen *Sclerotinia sclerotiorum* self-organize spatially with different genetic programs activated along hyphae to promote virulence [3], highlighting the spatial heterogeneity of molecular interactions in this pathosystem. Single-cell and spatial transcriptomics revealed that plant immune responses exhibit cell-type-specific transcriptional differences [1, 4–7], opening new questions on how cell-specific immune responses are generated and how the activity of individual cells is integrated into robust disease resistance phenotypes within entire tissues.

Plants lacking specialized immune cells, efficient immune responses require the concerted reprogramming of cellular transcriptomes at the site of infection and systemically [1]. At the site of microbe entry, perception of the pathogen occurs either at the plant cell surface by pattern recognition receptors or intracellularly thanks to nucleotide-binding leucine-rich repeat receptors [8, 9]. Some plant immune responses are restricted spatially to a few cell neighborhoods. One such mechanism is the hypersensitive response, a rapid programmed cell death which prevents the spread of pathogens requiring living host cells to grow [10]. The spatial extent of the hypersensitive response is under genetic control as evidenced by mutants altered in regulators that exhibit runaway cell death phenotypes, in which cell death propagates beyond infected tissues [11]. Local immune responses at the site of pathogen infection trigger the production of signals that travel throughout the plant to activate systemic acquired resistance (SAR). SAR provides enhanced resistance against future pathogen attacks in uninfected, distal parts of the plant [12]. Pathogen perception by immune receptors initiates signal transduction cascades involving calcium influx, the activation of receptor-like cytoplasmic kinases, calcium-dependent protein kinases (CPKs) and mitogen-activated protein kinases, and the production of reactive oxygen species (ROS) [13–15]. Phytohormones such as salicylic acid (SA) and jasmonic acid (JA) act as cell-to-cell mobile signals enabling the activation and modulation of transcriptional defense responses [16]. ROS and calcium (Ca^2+^) waves are also considered important signals involved in both local and potentially rapid long-distance signaling [15, 17]. Phytocytokines, small signaling peptides, act as danger signals and are important for transmitting immune and damage responses between cells and tissues [12, 18]. These systemic signals are then perceived in distal cells, activating signaling and transcriptional responses away from the infection site. In addition to their role in cell-to-cell signaling, ROS and Ca^2+^ can modulate the activity of transcription factors (TFs) either directly or via interaction with sensor proteins such as calmodulin [17, 19]. Immune signal transduction pathways trigger extensive transcriptional reprogramming, with TFs serving as key sites for signal convergence. These responses are under tight regulation to adjust to multiple biotic and abiotic stresses in natural environments and to coordinate stress responses with growth and development [20, 21]. TFs associate with co-regulators, DNA and RNA modifiers, chromatin remodelers and post-transcriptional modifiers such as kinases, proteases, SUMO/ubiquitin, and second messengers like ROS and calcium ions to form regulatory networks essential for immunity [22, 23]. The precise mechanisms by which various cell types consistently switch their transcription towards immune responses remains however largely elusive.

Cellular heterogeneity in gene expression is often referred to as transcriptional noise [24]. Noise can maintain phenotypic plasticity, enabling cells to adjust to external cues, differentiate into specialized cell types and states, and mitigate the impact of cellular malfunctions [24–26]. However, uncontrolled variability can disrupt tissue function, lead to loss of cell identity, and contribute to diseases like cancer [24] so that multiple biological mechanisms have been suggested to limit the detrimental effect of gene expression variability. Variability in transcript abundance is caused primarily by cellular intrinsic and extrinsic sources. Intrinsic noise originates from the stochastic nature of biochemical reactions involved in transcription, leading genes to switch randomly between “on” and “off” states, resulting in fluctuations in mRNA levels over time and across cells [27, 28]. Extrinsic noise originates from upstream variations in the cellular state that affect the expression of potentially multiple genes simultaneously such as cell cycle stage, cell size and shape, microenvironment of the cells, including concentration in regulatory molecules [28, 29]. This initial variability is then subject to modulation by gene-specific features such as promoter architecture and epigenetic states [24, 25], regulatory networks, and post-transcriptional processes like nuclear export and mRNA degradation, which shape the final distribution of mRNA observed in the cytoplasm. In several systems, cytoplasmic transcript variability is largely explained by deterministic cell state differences rather than purely stochastic bursting [28, 30], the contribution of each source can vary depending on the gene, cell type, and environmental conditions [24]. At the tissue level, the coordinated response to external signals can involve modulating the fraction of cells that switch to a transcriptionally active state, rather than modulating the transcription rate across all cells in a graded manner [31]. In mammals, a synergy between intrinsic noise-mediated oscillation and robustness conferred by extrinsic noise could enable more efficient gene regulation by the NF-κB signaling system in unstable environments [32]. Rosales-Alvarez et al. [33] proposed that immune responses in murine hematopoietic cells could be regulated by “noisy switches” prone to intrinsic noise, that would enable a broad spectrum of responses across a population of cells, followed by clonal expansion of cells with the appropriate response level. Whether the regulation of transcriptional noise contributes to tissue-level immune responses in plants remains currently unknown.

To document transcriptional responses at the cellular level, we combined end-point single-nucleus RNA-sequencing (snRNA-seq) with time-course 3’ tag-based RNA-sequencing (tagseq) [34] of *Arabidopsis thaliana* leaves inoculated with *S. sclerotiorum*. We analyzed the nuclear transcriptome of 7 279 leaf cells supporting extensive heterogeneity of plant immune responses across cell types, and in sub-populations of mesophyll cells. Correlation of pseudotime trajectories with tagseq experimental time-course of expression allowed to reconstruct and calibrate the spatiotemporal dynamics of single cell responses to fungal inoculation. Taking advantage of snRNA-seq, less prone to cytoplasmic buffering of transcriptional noise [33], we reveal an overall increase in extrinsic transcriptional noise and a decrease in intrinsic transcriptional noise in nuclei from inoculated cells. Changes to transcriptional noise patterns were stronger in nuclei close to the infection site, and targeted primarily genes associated with Ca^2+^ signaling and immune functions. Using the R-GECO1 cytoplasmic Ca^2+^ reporter [35], we found that the intensity of Ca^2+^ variations upon *S. sclerotiorum* inoculation coincides with variations to transcriptional noise in space and time, suggesting a functional link between these cellular responses. This work establishes a previously unknown relationship between transcriptional noise and the coordination of immune responses in plants, using calcium as an intercellular relay. The gene expression dataset generated from this study will support in-depth investigations of the molecular bases of diseases caused by necrotrophic fungi. Knowledge gained from our results shed new light on the mechanisms underlying plasticity and robustness of plant immune responses, that can inform the design of strategies to reduce pathogen damage to crops in unstable environments.

## Results

### Construction of an *Arabidopsis* leaf nuclear transcriptome atlas during *S. sclerotiorum* infection

**Fig. 1:**
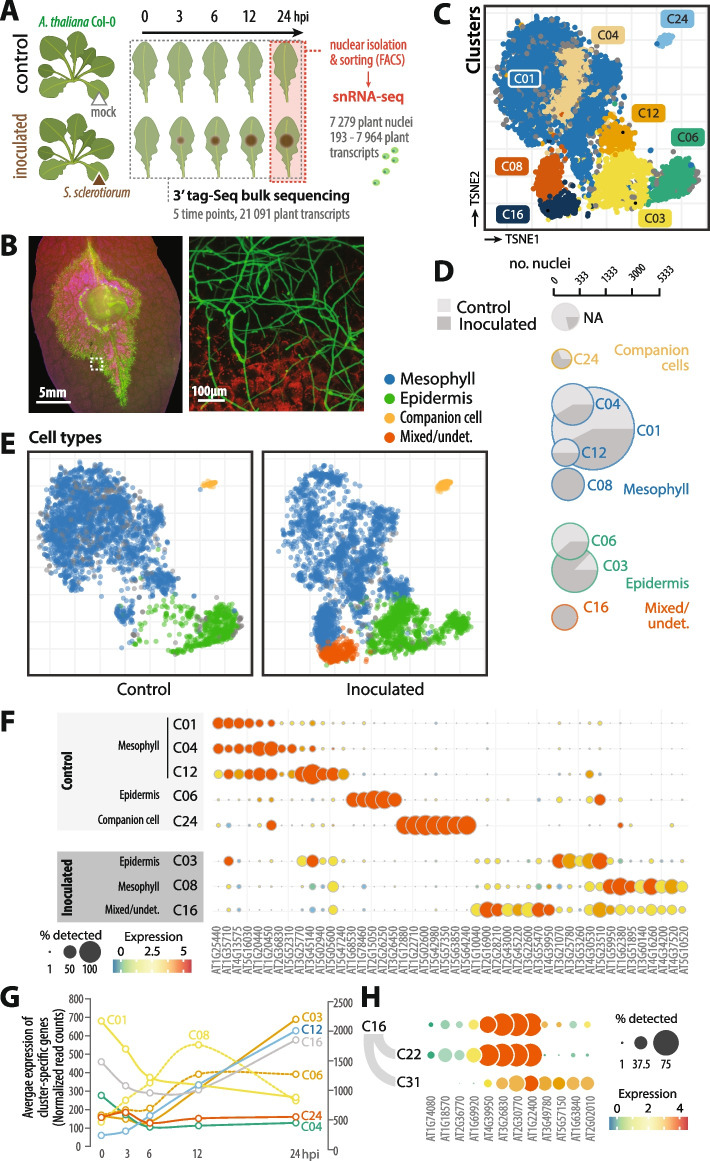
A transcriptome atlas of *Arabidopsis* leaf response to *S. sclerotiorum* based on single nucleus and bulk time course RNA sequencing. **A** Schematic overview of the sampling and transcriptome analysis strategy used in this work. Triangles indicate inoculation points. **B** Representative fluorescence pictures of *A. thaliana* Col-0 inoculated by a *S. sclerotiorum* strain expressing GFP at 24 h post inoculation (hpi). The dotted square indicates the approximate position of the region imaged in the micrograph shown. **C** A transcriptome atlas of 7 279 *A. thaliana* nuclei shown as t-distributed stochastic neighbour embedding plot (t-SNE) projection of the single nucleus RNA-seq data in two dimensions. Each point represents a nucleus, inter-point distances indicate the degree of similarity over all principal components and colours distinguish the coarsest level (level 1) clusters from Leiden clustering labelled C01 to C24. Nuclei in grey we not confidently assigned to any cluster. **D** Size and proportion of inoculated nuclei in each level 1 cluster. The size of circles indicates the total number of nuclei per cluster, with the proportion of control and inoculated nuclei shown as pie sectors. Cell type assignment for each cluster is labelled and indicated by a color code. NA, not attributed. **E** Separate t-SNE plots for control and inoculated samples showing cell type assignment for each nucleus. **F** Selected marker genes associated with cell-type and resistance responses for each cluster. Gene expression is shown for control nuclei except for C03, C08 and C16 in which inoculated nuclei make > 85% of the cluster. Circle sizes indicate the proportion of nuclei within each cluster for which a gene is detected, and colour indicates the average normalised log count (expression). **G** Average expression (Normalized read counts) of cluster-specific genes in the tagseq time course. C01 is plotted along the rightmost Y-axis, all other clusters along the leftmost Y-axis. **H** Selected marker genes associated with sub-clusters C22 and C31 of level 1 cluster C16. Circle sizes indicate the proportion of nuclei within each cluster for which a gene is detected, and colour indicates the average normalised log count (expression). Undet., undetermined

To study transcriptional heterogeneity across cells during plant immune responses, we performed single-nucleus RNA-sequencing of mock-treated and fungal pathogen-inoculated *A. thaliana* leaves (Fig. 1A, B). The output and unique molecular identifier (UMI) clustering from alevin-fry [36] yielded 272 817 nuclear barcodes, from which 7 279 (3 327 infected, 3 952 non-infected) candidate nuclei were retained after filtering to remove possible empty or doublet droplets, low read coverage, and excessive organelle content (Additional file 1: Fig. S1). Read pair numbers per nucleus ranged from 204 to 39 667, with a median of 962. The number of genes detected per nucleus ranged from 193 to 7 964, with a median of 590 and mean of 886 genes. Genes were detected in between 0 and 99% of nuclei, with a median of 0.8% and a mean of 3% of nuclei. The mean gene read pair counts per nucleus ranged from 0 to 55, with a median of 0.01.

To summarize the main patterns of variation, we decomposed normalized counts using principal component analysis (PCA). Principal components (PCs) 1 and 2 explained 9.2% and 4.5% of the total variance respectively. The top 25 PCs explaining 38% of the total variation were used in all subsequent analyses, after removing potential batch effects using HARMONY [37]. To identify nuclei populations, we clustered nuclei from Harmony corrected PCs using Leiden clustering, and classified nuclei independently of PCA using consensus non-negative matrix factorization (CNMF). Because the Leiden algorithm is sensitive to random seeds, we used multiple random seeds to construct consensus clusters, and identified consistent hierarchical clusters at 3 levels, each representing increasing granularity of cell groups (Additional file 1: Fig. S2). The coarsest level included 8 nuclei clusters (Fig. 1C, D, Additional file 2: Table S1) that could be further subdivided down to 35 clusters at level 3 (Additional file 1: Figs. S3, S4, Additional file 2: Table S2). We observed a good correspondence between CNMF factors and predicted clusters, indicating that the observed groupings of nuclei are robust, and that both CNMF and hierarchical clustering capture substructure and resistance relevant information (Additional file 1: Fig. S5, Additional file 2: Tables S3, S4). For convenience, we mostly focus on level 1 clustering in further analyses to highlight infection responses.

To manually assign the best candidate cell type and state per cluster, we used published cell-specific markers and enrichment tests of curated bulk RNAseq and snRNA-seq samples in the scPlantDB [38] and PCMDB [39] databases (Additional file 2: Tables S5-S7). Five clusters (C01, C04, C06, C12 and C24) had a relatively balanced proportion of control and inoculated nuclei, with 39% to 69% of inoculated nuclei per cluster, while clusters C03, C16 and C08 were largely dominated by inoculated nuclei (87.2%, 99.4% and 100% respectively, Fig. 1D, Additional file 2: Table S1). At hierarchical level 1, major leaf cell types were identified including four clusters (C01, C04, C08 and C12) classified as mesophyll, two clusters (C03 and C06) classified as epidermis, one cluster (C24) classified as companion cells and one cluster (C16) of undetermined identity (Fig. 1E, F). Mesophyll clusters were identified based on enrichment of gene ontology (GO) terms involving chloroplasts and photosynthesis and mesophyll associated markers such as *BBX15* (*AT1G25440*), *AT4G13575*, *AT5G16030* (C01, C04, C12), *PEC1* (*AT5G02940*) and *LOX2* (*AT3G45140*) (C12), *BGLU30* (*AT3G60140*) and *AT4G16260* (C08). Epidermal clusters were identified based on specific markers including *CER6* (*AT1G68530*) and *KCS10* (*AT2G26250*) for C06, *NADK1* (*AT3G21070*) and *AT5G23510* for C03. Cluster C24 was annotated as a companion cells as it contained markers for phloem/companion cells including *SUC2* (*AT1G22710*), *HMP42* (*AT5G02600*) and *AHA3* (*AT5G57350*). Finally, cluster C16 showed specific expression of mesophyll-associated genes *PME17* (*AT2G45220*) and *LTPG5* (*AT3G22600*) but also epidermis-associated genes *ACA2* (*AT2G28210*) and *JUB1* (*AT1G10040*). Based on this we suggest that C16 clustering is primarily determined by pathogen response and the cluster contains a mixture of both epidermal and mesophyll nuclei [7]. To get insights into the relative contribution of cell clusters to *Arabidopsis* response to *S. sclerotiorum*, we analyzed the average expression of cluster-specific genes in a tagseq time course of expression (Fig. 1B, G). Consistent with the proportion of inoculated nuclei in each cluster (Fig. 1D), early time points were dominated by the expression of specific genes from C01 and C04. The end of the time course was dominated by specific transcripts from C03, C12 and C16. Genes specific from C08 peaked at 12 h post inoculation (hpi). This suggests that, in mesophyll cells, C01 and C04 could harbor constitutive and early defenses, C08 could form intermediate responses, while C12 and C16 could correspond to late responses. Although limited to cluster-specific genes, this overview recapitulates the probable sequence of cell-specific responses to fungal pathogen inoculation.

Clusters at higher granularity recovered more specific cell types, such as C13 (level 2, sub-cluster of C01) which is enriched in “bundle sheath” and “xylem” DEGs from scPlantDB and PCMDB, including *GSM1* (*AT5G23010*) and *MYB28* (*AT5G61420*) (Additional file 1: Figs. S2, S4). Cluster C16 could be further subdivided into C22 and C31, that we characterized using discriminating marker genes (Fig. 1H). The expression of *MYB51* (*AT1G18570*) and *MYB122* (*AT1G74080*), associated to fungal pathogen response in vasculature and epidermal cells respectively [7], was largely restricted to C22 nuclei. In agreement with MYB51 activity, C22 also strongly enriched in glucosinolate metabolism-related genes such as *CYP71A13* (*AT2G30770*), *GSTU12* (*AT1G69920*) and *PAD3* (*AT3G26830*). Nuclei from C31 specifically expressed *PSK4* (*AT3G49780*), associated with guard cells and mesophyll in scPlantDB, *GAD4* (*AT2G02010*) mediating gamma-aminobutyric acid biosynthesis (GABA) and systemic defense responses [40]. This suggests that local responses involving phytoalexin biosynthesis, and systemic signaling by phytosulfokines and GABA are mediated by distinct cell populations, highlighting complementary cell activity in defense against *S. sclerotiorum*.

Our results indicate that transcriptome signatures and nucleus populations were characterized by cell type and responses to fungal infection. We generated a single-nucleus transcriptome atlas that covered major cell types in *Arabidopsis* leaf tissues, and captured a snapshot of the heterogeneity in gene expression changes during necrotrophic infection by a fungal pathogen.

### Ubiquitous and cell population-specific responses to *S. sclerotiorum*

**Fig. 2:**
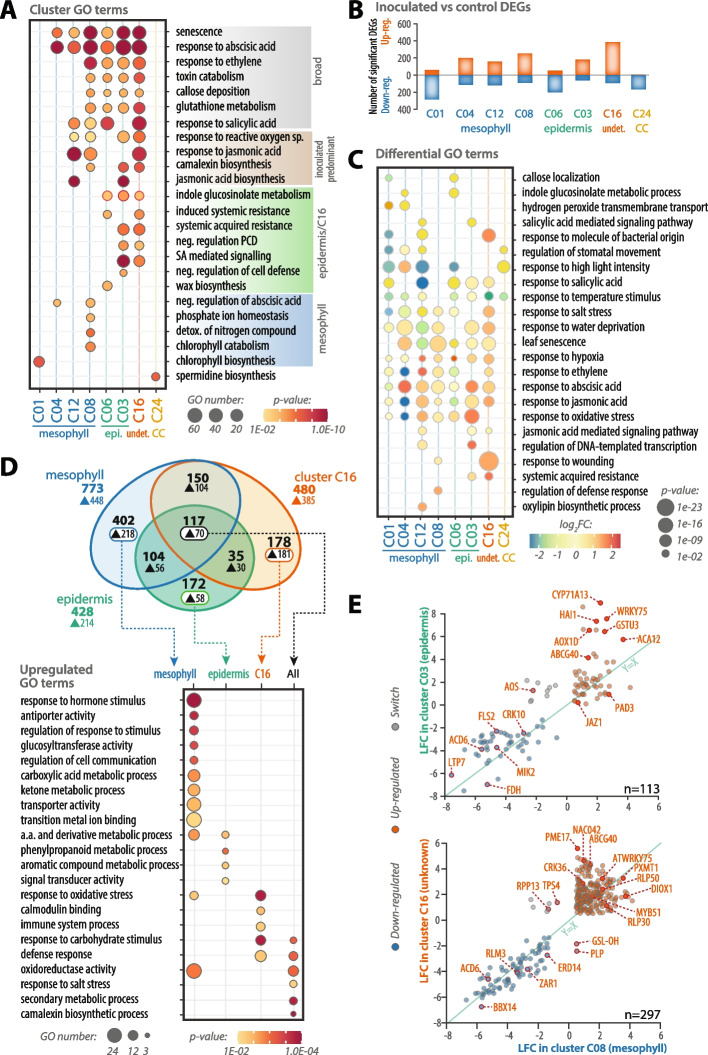
Characterization of pathogen responses in single nucleus clusters. **A** Abundance and enrichment *p*-values of gene ontologies (GO) in single nucleus clusters. Circle sizes indicate the number of genes upregulated in inoculated nuclei harboring selected enriched GO in each cluster, with colors indicating the enrichment *p*-value from a Fisher exact test. **B** Distribution of up- and down-regulated genes per single nucleus cluster. Differential expression was determined based on the comparison of genes expression between inoculated and control nuclei within each cluster, except for C08 and C16 for which nuclei of all other clusters were used due to lack of control nuclei within these clusters. **C** Differential expression analysis of GO terms in control versus inoculated nuclei. The size of circles indicates differential expression *p*-value and colors show log2 fold change (FC) of expression. **D** Distribution of differentially expressed genes (DEGs) across major cell types identified. Number of DEGs are indicated in bold, number of up-regulated genes indicated by a triangle (▲). The bottom diagram shows abundance and enrichment *p*-values of gene ontologies (GO) per cell types among genes up-regulated specifically in one cell type or in all three cell types, highlighted by a white box in the venn diagram. **E** Pairwise comparison of gene LFC in clusters C03, C08 and C16 dominated by inoculated nuclei. n indicates the number of shared DEGs in each comparison. Selected genes are labelled. Cluster labels are colored according to cell type in blue (mesophyll), green (epi., epidermis), yellow (CC, companion cells) and red (undet., undetermined or mixed cell types). A.a, amino-acid; detox., detoxification; neg., negative; sp., species; PCD, programmed cell death; SA, salicylic acid

To document the diversity of cell population responses to *S. sclerotiorum* inoculation in *A. thaliana*, we analysed GO enrichment and differential gene expression. First, we analysed GO enriched in genes expressed in each nucleus cluster, relative to all expressed genes (Fig. 2A, Additional file 1: Fig. S6, Additional file 2: Table S8). GO terms senescence, response to abscisic acid, response to ethylene, toxin catabolism, callose deposition, gluthatione metabolism, and response to SA were enriched in mesophyll, epidermis and C16 clusters, forming a response broadly distributed across cell types and populations. GO terms response to ROS, response to JA, camalexin biosynthesis and JA biosynthesis were restricted to clusters in which inoculated nuclei dominate (C12, C08, C03 and C16), probably representing mid to late responses. GOs indole glucosinolate metabolism, induced systemic resistance, systemic acquired resistance, negative regulation of programmed cell death, SA-mediated signaling, negative regulation of cell defense, and wax biosynthesis were enriched in epidermis or C16 clusters but not in mesophyll clusters. Conversely, negative regulation of abscisic acid, phosphate homeostasis, detoxification of nitrogen compound, chlorophyll catabolism and biosynthesis were only enriched in mesophyll clusters (C01, C04, C12 and C08). Finally, cluster C24 was enriched in GOs including spermidine and arginine biosynthesis, sucrose metabolism, phloem glucosinolate loading, and negative regulation of H_2_O_2_ biosynthesis. Interestingly, clusters enriched in biosynthesis and response to JA only partly overlapped (only C12 has both terms enriched). Similarly, clusters enriched in SA signaling and response were partly distinct (only C16 had both terms enriched).

Second, using single nucleus transcriptome atlases created from the control and inoculated samples, we identified differentially expressed genes (DEGs) between control and inoculated samples. C08 and C16 lacking control nuclei, we used gene expression in other clusters as reference to call DEGs in these clusters. This identified 305 significant DEGs per cluster in average, yielding 1 266 non-redundant significant DEGs across all clusters, with an up/down-regulated ratio 1.64 in average (Fig. 2B, Additional file 2: Tables S1, S9). Differential analysis between inoculated and mock samples at each time point of our tagseq dataset identified 8755 DEGs (|LFC|≥ 1, adj. p-val ≤ 0.01, Additional file 2: Tables S10-S11). The two RNA-seq strategies were largely consistent, yielding 948 DEGs in common (74.9% of the snRNA-seq DEGs), but also revealed specific sets of DEGs. Notably 318 DEGs were only detected by snRNA-seq, including 73 genes up-regulated upon inoculation. Up-regulated genes detected only in the snRNA-seq included notably the receptor-like protein *RLP50* (*AT4G13920*), the calmodulin binding-like-interacting protein kinase *CIPK11* (*AT2G30360*), the disease resistance genes *RPP13* (*AT1G59218*) and *LAZ5* (*AT5G44870*), each differential in one or two single nuclei clusters only. Consistent with previous bulk RNA-seq reports [21], tagseq identified 4 095 up-regulated genes, corresponding to ~ 45% of DEGs. The specific limits of single nuclei and bulk RNA-sequencing highlight the importance of combining single cell and whole tissue transcriptomics for a more complete understanding of pathogen responses in plants [7].

To document biological processes regulated by inoculation in each cluster, we performed differential analysis at the GO level (Fig. 2C, Additional file 1: Figs. S6, S7). Callose localisation, indole glucosinolate metabolism, H_2_O_2_ transmembrane transport and SA-mediated signalling were each down-regulated specifically in two clusters among C01, C04, C06 and C12. Multiple differential GOs were shared across three to seven clusters, but all showed contrasted regulation patterns. For instance, response to SA was upregulated in C03, C12, and C16, but down-regulated in C01 and C06. Response to JA was upregulated in C03, C04, C08, C12, and C16, but downregulated in C01 and C06. Regulation of transcription, response to wounding, SAR, regulation of defense and oxylipin biosynthesis were upregulated specifically in one or two clusters among C03, C08, C12, and C16. Cluster-specific GO enrichment and contrasted regulation patterns across clusters support cell population-specific regulation of responses to *S. sclerotiorum*.

To distinguish core and specific pathogen responses at the cell type level, we compared DEGs detected in mesophyll clusters (C01, C04, C08 and C13), epidermal clusters (C03 and C06), and in C16 (Fig. 2D). We identified a total of 770 DEGs in mesophyll cells, 428 in epidermal cells, and 480 in C16 cells, yielding 1 158 unique DEGs across all cell types. Of these, 717 genes were significantly up-regulated. Notably, 117 DEGs (10.1%) were shared among all three cell types, including 70 genes (9.1%) that were significantly up-regulated. This overlap suggests the existence of a reduced core pathogen response program, although its extent may be underestimated due to technical constraints, such as limited sequencing depth and suboptimal nucleus extraction efficiency. To investigate the functional specificity of these responses, we performed GO enrichment analysis on up-regulated genes, both within cell type-specific sets and the shared core set. The resulting GO term distribution revealed complementary signalling functions among the three cell types (Additional file 2: Table S12). Mesophyll cells were characterized by an enrichment of hormone response pathways, whereas epidermal cells exhibited increased signal transducer activity, and C16 cells showed enrichment for calmodulin binding and carbohydrate response pathways. Further specialization was observed in metabolic processes: mesophyll cells preferentially activated pathways associated with carboxylic acid and ketone metabolism, while the epidermis was enriched in genes involved in phenylpropanoid metabolism. Additionally, mesophyll cells showed significant enrichment for GO terms related to cell communication and transport processes, indicating a potential central role in integrating cellular responses across tissue types.

To contrast cell responses to pathogen in a quantitative manner, we compared log_2_ fold changes (LFCs) for DEGs in clusters C03, C08 and C16, dominated by inoculated nuclei (Fig. 2E). We observed a strong overall correlation in LFC between clusters C03 and C08 (*R*^2^ = 0.72), and between C08 and C16 (*R*^2^ = 0.78), primarily driven by consistently down-regulated genes and weakly up-regulated genes that exhibited similar expression trends across clusters. Despite this general concordance, a subset of genes displayed marked quantitative differences in expression. Specifically, 37 and 47 genes exhibited LFC variation (ΔLFC) ≥ 2 in the C08–C03 and C08–C16 comparisons, respectively, representing 32.7% and 15.8% of the DEGs analysed in each case. Among these, *CYP71A13* (*AT2G30770*) showed a striking > 100-fold difference in expression between C08 and C03 (ΔLFC = 6.74), while *PME17* (*AT2G45220*) exhibited a > 30-fold difference between C08 and C16 nuclei (ΔLFC = 6.74). Furthermore, 17 genes switched between up- and down-regulated in cluster comparisons. For instance, the *TPS4* (*AT1G61120*) terpene synthase was up-regulated in C16 (LFC 1.4, p-val 3.4E-55) but down-regulated in C08 (LFC −0.74, p-val 1.8E-22). These findings exemplify pronounced quantitative variation in gene regulation in response to pathogen inoculation, suggesting that expression responses to *S. sclerotiorum* are not uniform across cell populations. Such heterogeneity may be attributed to factors including cell-type-specific regulatory mechanisms, differential timing of the response, or spatial variation in proximity to the pathogen.

### Single nucleus populations capture various stages of *Arabidopsis* responses to *S. sclerotiorum*

Fungal infection is a spatiotemporal dynamic process in which signals are exchanged between cells, and subsets of plant cells come progressively in contact with the pathogen as it colonizes plant tissues. We observed a general trend in the t-SNE plots and clustering for three possible cell trajectories, distinguishing epidermal (C01 > C03 > C06), vascular tissue (C01 > L2C13 > C24), and resistance response (C01 > C08 > C16) nuclei (Additional file 1: Fig. S2) that may represent temporal stages in the infection process.

**Fig. 3:**
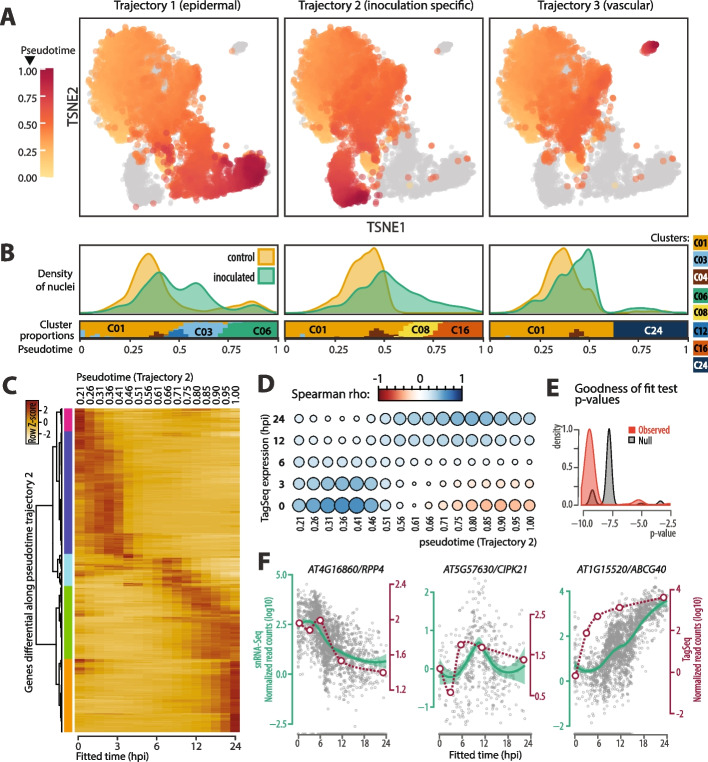
Pseudotime analyses reveal putative spatio-temporal trajectories of pathogen responses. **A** t-SNE projection of single nuclei colored according to pseudotime assignment along three dominant trajectories. Trajectory 2 is consistent with an increase in the proportion of inoculated nuclei over time and therefore represents the most likely temporal dynamics of nuclear transcriptome following pathogen inoculation. **B** Distribution of nuclei along pseudotime trajectories, showing the density of control and inoculated nuclei and the proportion of nuclei from each cluster (control and inoculated). **C** Heatmap showing the expression of genes differential along pseudotime trajectory 2. **D** correlation between tagseq time-course and pseudotime trajectory 2 for the expression of genes differentially expressed along pseudotime trajectory 2. **E** Distribution of *p*-values for goodness-of-fit tests between the expression of genes differentially expressed along pseudotime trajectory 2 in tagseq and snRNA-seq. Observed *p*-values are shown in red, the null distribution resulting from 10 000 shuffling of time points shown in grey. **F** Expression patterns of representative early-, mid- and late-peaking genes along pseudotime trajectory 2. Grey dots represent gene expression in individual nuclei, with loess local regression and 95% confidence intervals shown in green (left Y-axis). Average normalized read counts obtained from tagseq are shown in red (right Y-axis)

To reconstruct the dynamics of nucleus transcriptome reprogramming upon inoculation, we performed trajectory analysis from the HARMONY corrected PCs using Slingshot [41]. We recovered the epidermal (lineage 1), pathogen response (lineage 2) and vascular tissue (lineage 3) trajectories (Fig. 3A, B). Similar trajectories were obtained by estimating the pseudotime trajectories for the mesophyll and epidermal subsets of our nuclei separately as performed by [1, 7] (Additional file 1: Fig. S8). In addition, this approach did not support a direct link between clusters C08 and C16 and either cluster C03 or C12. C03 and C12 did not cluster well with either the mesophyll or epidermal subsets, but remained more similar to each other than any other cluster. They may therefore represent an additional distinct infection response cluster or trajectory endpoint.

To pinpoint genes contributing to transcriptome reprogramming along these trajectories, we used a generalized additive model implemented in the tradeSeq R package to test for genes with different expression patterns along each pseudotime trajectory and between infected and non-infected nuclei along trajectories [42]. We identified 583, 666, and 519 genes with significant time-dependent expression patterns (Wald Test, Benjamini–Hochberg adjusted *p*-value < 0.01) at any point along the epidermal, resistance response and vascular trajectories, respectively (Fig. 3C, Additional file 2: Tables S13-S15).

To test whether pseudotime trajectories recapitulated dynamic transcriptome reprogramming, and to calibrate these trajectories, we compared regulation patterns along pseudotime with those obtained in our tagseq time course. Global correlation for genes significantly regulated along pseudotime trajectories supported a good agreement in the transcriptome reprogramming along pseudotime ~ 0.21 to 1 and tagseq expression between 0 and 24 hpi (Fig. 3D, Additional file 1: Fig. S9). Timepoint 6 hpi in the tagseq experiment and pseudotime points ~ 0.50 to ~ 0.60 generally showed weaker correlation across experiment, suggesting they represent a turning point where spatio-temporal patterns of gene expression are changing rapidly or locally. To further support the correspondence between tagseq kinetics and pseudotime, we performed goodness-of-fit tests between these two datasets for each gene showing time-dependent expression (Fig. 3E). *P*-values for observed were generally ~ 100-fold lower than those for a null distribution, supporting non-random association between pseudotime and the experimental expression time course.

Confronting pseudotime analysis with localization of a fluorescent reporter and microdissection data, Tang et al. [7] proposed that pseudotime trajectories may reflect the relative position of a cell towards fungal pathogens. Along our trajectory 2, early-peaking genes included known pathogen-perception genes such as *RPP4* (Fig. 3F), *RLM3*, *MIK2* and *SOBIR1*, mid-peaking genes included known signaling genes such as *CIPK21* (Fig. 3F), *WRKY33* and *ANAC19*, late-peaking genes included defense response genes such as *ABCG40* (Fig. 3F), *PAD3* and *JAZ1*, recapitulating the sequence of key steps in plant immune responses. This sequence of gene activation along pseudotime is consistent with relative proximity of nuclei to the fungus increasing along trajectory 2.

### Fungal inoculation increases extrinsic transcriptional noise in immunity and signaling-related genes

**Fig. 4:**
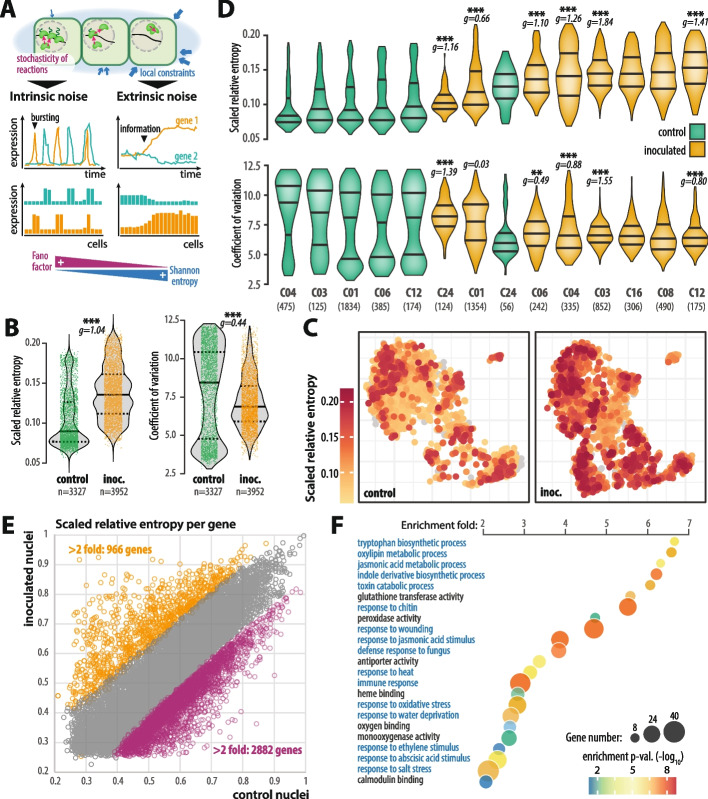
Pathogen inoculation triggers a shift in intrinsic and extrinsic transcriptional noise patterns. **A** Schematic representation of types and sources of transcriptional noise, and their impact on gene transcription. Pink arrows illustrate alternative interactions of DNA molecules with RNA-polymerase complexes, underlying stochasticity in the transcription process. Blue arrows illustrate variable external constraints at the cell level. Line and bar plots illustrate the potential effect of noise on transcription at the cell level and the cell population level respectively. **B** Distribution of Shannon entropy and Fano factor for gene expression per nucleus in control (green) and inoculated (yellow, inoc.) nuclei from our snRNA-seq atlas. Kernel probability of the data is shown in grey with individual datapoints. Median is shown as thick plain line, 1 st and 3rd quartile values as dotted lines. Effect size estimated by Hedge’s g and the significance of a Wilcoxon rank sum test for the difference between control and inoculated samples is shown on graphs (*p*-value: *** < 0.01, ** < 0.05). **C** Distribution of Shannon entropy and Fano factor for gene expression per nucleus in control (green) and inoculated (yellow, inoc.) nuclei for each nucleus cluster, following the same representation as in B. Value between brackets indicates the number of nuclei per cluster. **D** T-SNE projection showing control and inoculated nuclei colored according to their Shannon entropy of gene expression. **E** Shannon entropy of individual gene expression across nuclei, in control (X-axis) and inoculated nuclei (Y-axis). Points corresponding to the 2 882 genes with > 2fold higher entropy in control nuclei relative to inoculated nuclei are shown in purple, points corresponding to the 966 genes with > 2fold higher entropy in inoculated nuclei relative to control nuclei are shown in yellow. **F** Enrichment analysis for gene ontologies (GOs) among the 966 genes with higher entropy in inoculated nuclei. The size of circles indicates the number of GO instances among the 966 genes, colored according to the enrichment *p*-values (hypergeometric test). Biological process GOs are labelled in blue, molecular function GOs in black

Single-cell and nucleus RNA sequencing gives access to the heterogeneity of gene expression in isogenic cells, a property generally referred to as transcriptional noise. The origin of noise is typically divided between intrinsic noise, resulting from fluctuations in biochemical reactions occurring during transcription and translation, and extrinsic noise resulting from the specific state and environment each individual cell is in. Intrinsic and extrinsic noise can have both stochastic and deterministic causes [24, 29] (Fig. 4A).

To evaluate the impact of fungal pathogen inoculation on transcriptional noise in plant cells, we quantified molecular variability in cell populations from healthy and pathogen-inoculated leaves. We used the Fano factor as a proxy for intrinsic transcriptional noise and Shannon entropy as a proxy for extrinsic transcriptional noise [24, 26, 43–45] (Additional file 1: Fig. S10, Additional file 2: Table S16).

Overall, inoculated nuclei showed significantly higher extrinsic noise than control nuclei (median 0.138 and 0.089 respectively, Hedge’s g = 1.04), but lower intrinsic noise than control nuclei (median 6.88 and 8.52 respectively, Hedge’s g = 0.44) (Fig. 4B). Control nuclei showed a biphasic distribution of intrinsic noise in which 32.7% and 27.4% of nuclei had a Fano factor > 10 and < 5 respectively (6.0% and 7.1% respectively for inoculated nuclei). With the exception of cluster C24, inoculated nuclei had a median extrinsic noise higher than control nuclei in all clusters, and clusters specific to inoculated treatment C08 and C16 ranked in the top 3 clusters with the highest median extrinsic noise (Fig. 4C, D, Additional file 1: Figs. S11, S12). The entropy increase in inoculated nuclei was significant for all clusters (Wilcoxon test *p*-value < 0.01) with medium effect size for C01 and large effect size for other clusters (Hedge’s g > 1). The intrinsic noise decrease in inoculated nuclei was significant for C06, C03 and C12, with effect size negligible for C01, small for C06, medium for C12 and large for C03 and C04. For all cell types, the amplitude of transcriptional noise variation upon inoculation increased with the proportion of inoculated nuclei per cluster. Assuming that pseudotime trajectory 2 reflects the relative distance between nuclei and the fungus, these results indicate that cells close to the fungus (C08, C16) undergo more drastic transcriptional noise variation than distal cells (C01).

To identify genes most affected by transcriptional noise variation upon inoculation, we calculated normalized Shannon entropy per gene across control and inoculated nuclei (Additional file 2: Table S17). This revealed 2 882 genes with > 2-fold decrease in entropy upon inoculation and 966 genes with > 2-fold increase in entropy upon inoculation (Fig. 4E). We then analyzed GO terms enriched in the 966 genes with clear entropy increase upon inoculation relative to the 15 269 genes detected in > 10 control and > 10 inoculated nuclei (Fig. 4F, Additional file 2: Table S18). The most enriched biological processes related to defense and stress response, including the biosynthesis of defense molecules such as oxylipins, JA and indole derivatives. Seven molecular functions were enriched in genes with increased entropy upon inoculation including glutathione transferase, peroxidase and antiporter activity, and heme, oxygen and calmodulin binding. Comparison between the list of genes showing increased entropy upon inoculation and DEGs indicated that many genes associated with response to fungus undergo an increase in entropy upon inoculation, such as response to JA, response to oxidative stress and indole derivatives biosynthesis (Additional file 2: Table S19, Additional file 1: Fig. S13). However, some pathogen response processes were less affected by entropy variation including cell wall modifications, cell death, response to SA and kinase-mediated signaling (Additional file 1: Fig. S13).

### Transcriptional noise and cytosolic calcium oscillations correlate in space and time upon pathogen inoculation

**Fig. 5:**
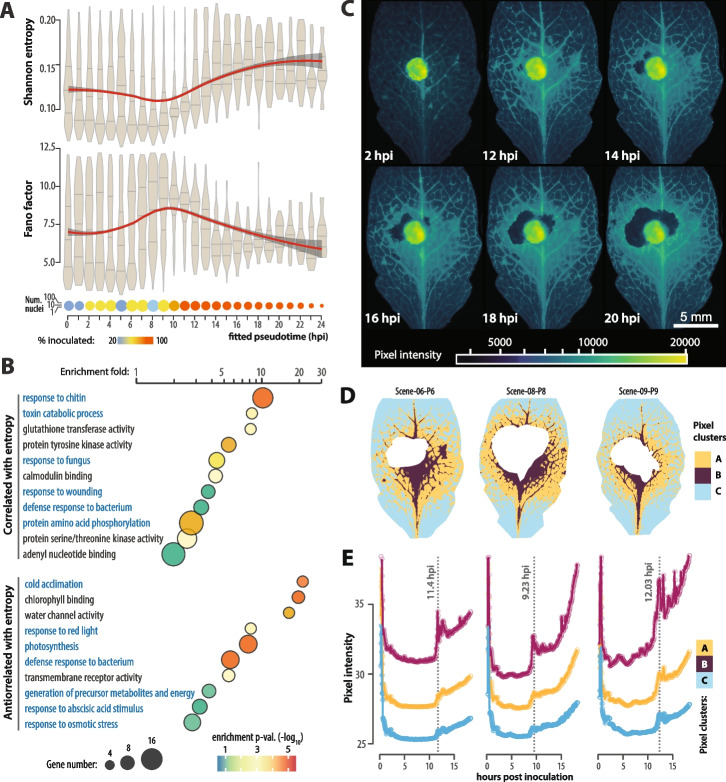
Co-regulation of transcriptional noise and cytoplasmic calcium spatio-temporal dynamics upon pathogen inoculation. **A** Distribution of Shannon entropy and Fano factor for gene expression in nuclei along pseudotime trajectory 2. Kernel probability of the data is shown in grey with median and quartile values shown as horizontal grey lines. Red lines show loess local regression, dark grey area shows and 95% confidence intervals. The bottom bubble plot shows number of nuclei (size of circles) in each pseudotime bin, with color indicating the proportion of inoculated nuclei per bin. **B** Enrichment analysis for gene ontologies (GOs) among genes with expression significantly correlated (156 genes) and anticorrelated (126 genes) with entropy along pseudotime trajectory 2. The size of circles indicates the number of GO instances, colored according to the enrichment *p*-values (hypergeometric test). Biological process GOs are labelled in blue, molecular function GOs in black. **C** Representative fluorescence pictures of an *A. thaliana* leaf expressing the R-GECO1 one reporter at six time points following *S. sclerotiorum* inoculation. The central bright circle corresponds to the agar plug used as inoculum. **D** Clusters of pixels resulting from the integration of R-GECO1 fluorescence over a 20 h time-course following *S. sclerotiorum* inoculation on three representative leaves. The central white area corresponds to dead cells at the end of imaging. **E** Quantification of R-GECO1 florescence over time in each pixel cluster for the three leaves shown in D. The vertical dotted lines indicate the beginning of calcium oscillations with time labelled. hpi, hours post inoculation

To document the temporal dynamics of transcriptional noise in *A. thaliana* following *S. sclerotiorum* inoculation, we analyzed Fano factor and Shannon entropy distribution along the pseudotime trajectory 2 (Fig. 5A). Entropy showed a biphasic pattern, with a slight overall decrease from median value 0.120 to a minimum median of 0.095 at 9 hpi, when inoculated nuclei became predominant along the pseudotime axis. Overall, entropy then increased 1.63-fold to reach median value 0.155 at 14 hpi, and remained roughly stable until the end of the time course. The Fano factor showed a mirror pattern with an increase from median 6.33 at 0 hpi to a peak 9.230 at 9 hpi, followed by a decrease to 6.23 at 14 hpi, and stable values until 24 hpi. These results indicate that a major global regulatory switch occurs between time points 9 and 14 hpi, involving a transient increase in intrinsic transcriptional noise followed by stabilization at a low intrinsic, high extrinsic transcriptional noise state.

To identify genes most strongly associated with variations of transcriptional noise following *S. sclerotiorum* inoculation, we performed a correlation analysis between transcriptional noise metrics and gene expression levels along pseudotime trajectory 2 (Additional file 2: Table S20). After Benjamini–Hochberg correction for multiple testing, 156 genes had an expression significantly correlated with transcriptional entropy (*ρ* > 0.64, adj.p-val < 0.01). Among the best correlated genes were *PAD3* (*AT3G26830*, *ρ* = 0.85), *BIK1* (*AT2G39660*, *ρ* = 0.8) and *CIPK23* (*AT1G30270*, *ρ* = 0.76). The biological processes ‘response to chitin’, ‘toxin catabolism’ and ‘response to fungus’ and molecular functions ‘glutathione transferase’, ‘protein tyrosine kinase’ and ‘calmodulin binding’ were the most enriched GOs among genes significantly correlated with entropy along trajectory 2 (Fig. 5B, Additional file 2: Table S21). There were 126 genes with an expression significantly anticorrelated with transcriptional entropy (*ρ* < −0.64, adj.p-val < 0.01) which were enriched in GOs ‘cold acclimation’, ‘chlorophyll binding’, ‘water channel activity’, ‘response to red light’ and ‘photosynthesis’. All clusters included DEGs with expression correlated or anticorrelated with entropy, their proportion was higher among DEGs in clusters C03, C08, C12 and C16 dominated by inoculated nuclei (Additional file 1: Fig. S13).

Cytosolic Ca^2+^ concentration coordinate multi-gene responses to dynamic external signal in yeast [46] and is a major signal in early plant immune responses [15, 35, 47]. The correlation between calmodulin binding genes expression and transcriptional noise variations prompted us to study the spatio-temporal dynamics of Ca^2+^ concentration following *S. sclerotiorum* inoculation. For this, we used lines expressing the R-GECO1 reporter and imaged Ca^2+^ concentration every 10 min for 24 hpi (Fig. 5C). We partitioned the leaf surface into three clusters according to the intensity and dynamics of calcium concentration per pixel (Fig. 5D). We detected a rapid and transient Ca^2+^ spike occurring between 9.5 and 12 hpi, which coincides with the onset of pathogen invasive growth (Fig. 5C, E). This spike gave rise to a Ca^2+^-enriched ring surrounding the mycelial colony, after which cytosolic Ca^2+^ concentration continued to increase gradually as the ring expanded outward around the advancing fungal front (Fig. 5C, E). The three spatio-temporal clusters defined concentric areas of Ca^2+^ dynamics. Cells located immediately adjacent to the lesion and in the vicinity of vascular cells (Cluster A) exhibited the highest Ca^2+^ concentrations and strongest temporal fluctuations (Fig. 5D, E). As the distance to the fungus increased, Ca^2+^ concentration decreased and oscillated less significantly (Clusters B and C), suggesting a localized and spatially-organized Ca^2+^ response to fungal invasion.

Cluster A represented 9–16% of the leaf surface, similar to inoculated nuclei cluster C16 dominating the end of pseudotime trajectory 2 (12.2%), cluster B represented 36–46% of the leaf surface, similar to inoculated nuclei clusters C04 and C08 dominating the middle of pseudotime trajectory 2 (32.9%), and cluster C represented 37–56% of the leaf area, similar to inoculated nuclei cluster C01 dominating the beginning of pseudotime trajectory 2 (53.8%) (Additional file 1: Fig. S14). These observations support a correspondence between pseudotime trajectory 2 and distance to the fungus. Together, our results suggest that cytosolic Ca^2+^ could be a signal triggering or propagating alterations to the regulation of transcriptional noise, enabling the systemic reprogramming of plant cells towards plant defense, including both generic and cell-type specific responses.

## Discussion

Single-cell methods have been used to dissect plant developmental processes with very high resolution [48–50] and more recently to the study of plant-pathogen interactions [1, 4–7]. Our snRNA-seq analysis provides a high-resolution view of the transcriptional landscape in *A. thaliana* leaves during infection with the fungal pathogen *S. sclerotiorum* and uncovers the cellular heterogeneity inherent in plant immune responses to necrotrophic fungi. Beyond the description of cell-specific responses distinguishing epidermal cells and sub-populations of mesophyll cells, we provide evidence that stochastic cell–cell variability is not simply the byproduct of molecular noise, but that unstable cell states play a key role in commitment to plant immunity. We report that an increase in intrinsic transcriptional noise coincides with bursts of cytosolic calcium during the plant’s response to fungal infection, and precedes the activation of key defense genes and the rise of extrinsic transcriptional noise in infected cells. This sequence of events would allow the exploration of new regions in the gene expression space [45, 51, 52], followed by a reconfiguration of the gene expression network into spatially organized immune responses. Such critical state transition might be a common signature in immune priming in plants.

A key challenge in studying plant-pathogen interactions lies in the uneven spatial distribution of pathogen invasion and colonization, particularly with filamentous pathogens, whose development within host tissues is highly dynamic [53]. We previously developed a macro-dissection approach to study plant and pathogen transcripts by RNA-seq and uncover spatial heterogeneity on the fungal side [3]. Bulk RNA-sequencing methods measure the average transcriptional profile across a population of cells, effectively masking the heterogeneity that single-cell approaches can reveal. In agreement, our differential analysis on snRNA-seq data identified 318 genes associated with pathogen response not detected by tagseq. Including genes differentially expressed along pseudotime trajectories (1 511 genes, Additional file 2: Tables S13-S15), our snRNA-seq experiment identified a total of 1 894 genes associated with cell-level responses to *S. sclerotiorum*, among which 501 (26.5%) were not detected by tagseq. Conversely, single-cell methods can be prone to lower sensitivity and confounding effects, leading to incomplete transcript capture [24, 29]. The use of single nucleus instead of single cell RNA-seq allowed reducing potential stress and biases induced by cell isolation [54] and importantly to bypass transcriptional noise buffering in the cytoplasm [28, 55–57]. In contrast to data obtained from protoplasts, snRNA-seq lacks access to cytoplasmic transcripts, typically resulting in fewer genes being detected per cell, which limits high-resolution cell-type annotation. Our study revealed specific responses and transcriptional noise patterns in the major leaf cell types involved in immune response to a fungal pathogen, but does not significantly extends knowledge to rare cell subtypes or transcripts.

We identified cells immediately surrounding the *S. sclerotiorum* infection site, corresponding nuclei cluster C16, that exhibit high cytoplasmic Ca^2+^ concentrations and significant fluctuations of transcriptional noise upon inoculation. This zone strongly correlates with the overstretched ring that emerges as a consequence of cell wall degradation by fungal hyphae, which relieves mechanical stress in the surrounding tissue. This mechanical release is known to activate mechanosignaling-triggered immunity (MTI), a mechanosensitive immune response mediated by the anisotropic reorganization of cortical microtubules [2]. Mechanical stimuli can directly trigger changes in cytoplasmic calcium concentration through the activation of various mechanosensitive ion channels localized in the plasma membrane and potentially intracellular membranes [58, 59]. The mechanically induced calcium signals are necessary for downstream responses such as changes to PIN1 auxin transporter polarity and the subsequent growth or organ initiation in the shoot apical meristem [58]. The spatiotemporal organization of cellular responses to *S. sclerotiorum* inoculation reported here may therefore be a downstream consequence of the initiation of MTI.

Transcriptional noise arises in part from stochastic events like transcriptional bursting and dynamic transcription factor binding [25, 31]. Ca^2+^ and ROS are critical signaling molecules involved in plant stress and defense responses [59]. Ca^2+^ signals, characterized by dynamic bursts and oscillations [35, 59, 60], can directly influence TF activity and localization. Ca^2+^-binding proteins such as calmodulin and CPKs, decode these signals, regulating TF function through mechanisms like phosphorylation/dephosphorylation. This can alter TF nuclear transport, stability, transactivation, and importantly, DNA binding affinity [19, 59]. Modified TF DNA binding affinity impacts gene expression patterns and can thus influences transcriptional noise [25, 51].

Studies in yeast provide a compelling model for how calcium signals can regulate TF activity dynamics, which in turn influences gene expression. In yeast, the transcription factor Crz1 translocates into the nucleus in response to calcium bursts. Strikingly, the external Ca^2+^ concentration controls the frequency, but not the duration, of these nuclear localization bursts [46, 60]. This “frequency-modulation” of TF activity is proposed as a general control strategy to coordinate multi-gene responses. By analogy, similar mechanisms could operate in plants, where variations in the frequency, amplitude, or duration of calcium spikes could encode information that influences the dynamic behavior of defense-related TFs. Variability in calcium signaling itself, arising from stochastic channel opening or variations in local calcium environments, could directly contribute to transcriptional noise by causing fluctuations in TF activity or nuclear localization. Furthermore, if different cells exhibit different calcium signatures in response to the same stimulus, this would naturally lead to cell-to-cell heterogeneity in the activation of calcium-regulated TFs and downstream gene expression. The spatially organized calcium response we observed during *S. sclerotiorum* infection, with higher concentrations and fluctuations closer to the lesion, suggests a spatially patterned signal that could drive localized differences in transcriptional regulation and noise.

Beyond direct TF interaction, evidence suggests that Ca^2+^ and ROS could regulate epigenetic marks [59, 61, 62]. Differences in CpG island composition at promoters have been linked to gene expression noise, suggesting that epigenetic features can be a source of variability [25, 29, 63–65]. The architecture of regulatory networks involving TFs, including feedback loops and indirect repression mechanisms, also influences noise levels [63, 66], and these networks are integrated with Ca^2+^ and ROS signaling pathways. Therefore, calcium signals, potentially integrated with other signals like ROS, could contribute to transcriptional noise by modulating TF activity, affecting promoter dynamics, or influencing the epigenetic landscape, thereby enabling the exploration of diverse defense transcriptional states. Future work will aim at testing causal links and temporal precedence in the connection discovered in this study between Ca^2+^, transcriptional noise switch and plant immune activation.

Transcriptional noise refers to the cell-to-cell variability in gene expression that exists within a population of genetically identical cells exposed to the same conditions [28]. Transcriptional noise in plants has both intrinsic (stochastic promoter dynamics, transcriptional bursts) and extrinsic (cellular regulatory pathways, cell state differences, population context, microenvironment) components. Factors influencing intrinsic noise include promoter architecture, the dynamics of TF binding, and chromatin state and modifications [24, 29]. Low numbers of molecules involved in transcription further contribute to stochasticity [29], suggesting that leaf cells may undergo a pause in transcription before commitment to defense. In several systems, including plants, extrinsic noise is often a dominant contributor to overall gene expression variability [25, 28, 29]. Our results indicate that while plants exhibit both intrinsic and extrinsic noise in gene expression generally [27, 31], the host response to pathogens, particularly in the immediate infection zone, is characterized by heightened extrinsic variability in defense-related gene expression. This could reflect heterogeneity in the timing, magnitude, or spatial distribution of individual cell responses to pathogen signals or mechanical stress. The enrichment of ontologies like calmodulin binding and peroxidase activity in these noisy genes suggests that signaling molecules like Ca^2+^ and ROS as potentially regulating noise through effects on TFs and epigenetics, are associated with this increased cell-to-cell variability in the defense response. Current data is however insufficient to determine whether Ca^2+^ acts as a signal triggering or propagating the transcriptional noise surge upon pathogen challenge. Future work will aim at characterizing the functional relationship between calcium oscillations and transcriptional noise in defense genes. Genetically-encoded noise reporters exploiting the MS2 tag [27] and *A. thaliana* calcium signaling mutants will be valuable resources to this end.

A longstanding model of cell fate determination proposed that differentiation starts with unstable transcriptome patterns, generating cell type diversity, upon which selection acts [51]. A testable prediction of this hypothesis is that an increase in gene expression heterogeneity should be observed during the critical phase of cell differentiation whenever the irreversible decision to commit is made. Studies in animal stem and progenitor cells support this view by showing a surge in transcriptional noise preceding irreversible commitment in a differentiation process [45, 67]. Differentiation can be conceptualized as a transition between stable states (attractors) of gene expression networks, and the increase in variability might facilitate cells overcoming the stability of the initial state to transition to a new differentiated state. Some evidence suggests that genome decompaction can lead to stochastic activation of gene expression as a first step toward fate commitment, potentially contributing to this surge in variability [68, 69]. Additionally, transcriptional memory, or the tendency for sister cells to have similar expression profiles, might be gradually erased during differentiation, contributing to increased variability at the clonal level and reconstituting the variability of the initial population.

In the context of plant defense against pathogens, the impact of transcriptional noise variation on host immunity remains to be formally assessed. The localized zone of increased transcriptional noise could represent cells exploring different defense states before committing to a specific response. Plant immunity involves a complex interplay of signaling pathways [23] that may lead to unstable states before stabilizing around the most appropriate defense responses. Cellular heterogeneity could allow for a population of cells to contain diverse transcriptional profiles, some of which may be pre-conditioned or better suited to respond effectively to the dynamic and spatially varying threats posed by the invading fungus and the associated mechanical cues [29]. For example, some cells might prioritize cell wall reinforcement, while others focus on producing antimicrobial compounds or activating programmed cell death. Quantitative resistance, which often involves multiple genes with small-to-moderate effects, is frequently associated with downstream defense mechanisms like the synthesis of defense metabolites or cell wall components, rather than just pathogen detection [70, 71]. Variability in the expression of these downstream defense genes within a cell population could provide a “bet-hedging” strategy, ensuring that at least some cells are prepared with the necessary molecular machinery to mount an effective defense [29]. This could be particularly advantageous against necrotrophic pathogens like *S. sclerotiorum*, which rapidly degrade host tissue and elicit complex stress responses [21, 72].

Furthermore, recent single-cell transcriptomics in plants has revealed that immune responses are not uniform across all cell types; distinct cell populations exhibit specific responses to infection [1, 7]. The localized transcriptional noise we observed could contribute to the emergence or fine-tuning of these cell-type-specific defense states, enabling a coordinated, yet heterogeneous, tissue-wide response. Our results showing cell-population specific regulation of responses to *S. sclerotiorum* support the outcome of such heterogeneous commitments. While some genes and biological processes were regulated similarly across several clusters, a majority of differentially expressed genes showed specific regulation in only one cluster. Furthermore, some GO terms like JA signaling, SAR, and oxylipin biosynthesis were specifically upregulated in subsets of clusters, while others like response to SA showed contrasted regulation patterns across different clusters, being upregulated in some and downregulated in others. Specific genes even showed a significant switch in regulation direction between clusters. This observed diversity in transcriptional responses across different cell populations within the infected tissue could reflect the successful commitment of cells within the noisy, mechanically perturbed zone to distinct, cell-type-specific defense programs required to contain the pathogen. In summary, a surge of extrinsic transcriptional noise may benefit plant immunity by promoting cellular heterogeneity to transiently explore diverse defense states, before stabilizing into specialized responses tailored to local micro-constraints.

In dynamically changing environments, cells utilize the synergy between oscillation and noise to achieve efficient gene expression [32, 73]. Signaling pathways can process fluctuating inputs by oscillating, providing the ability to decode frequency content [74]. Periodic signals and external cues can entrain these oscillations, increasing their amplitude and synchronizing gene output [75, 76]. Intrinsic noise within individual cells enhances oscillation and entrainment, leading to higher and coherent transcriptional output. Simultaneously, extrinsic noise, or cell-to-cell variability, broadens the range of inputs the population can effectively respond to, creating population robustness. This coordinated interplay allows for robust information processing and adaptation to diverse dynamic cues. In plant immunity, where rapid, dynamic transcriptional reprogramming is critical for responding to environmental fluctuations and pathogen attack, synergy between oscillatory signals such as Ca^2+^ and transcriptional noise might allow plant tissues to orchestrate a flexible yet robust defense response. This could manifest through mechanisms like modulating the fraction of cells actively transcribing or enabling ‘bet-hedging’ strategies within the cell population to cope with unpredictable pathogen challenges [29, 77, 78]. Our data suggests that the noise switch is not restricted to a specific cell type but primarily determined by proximity to the pathogen. Experiments using noise reporters would be required to map accurately the dynamics of the noise surge we report in this study.

## Conclusions

In summary, a local and transient switch in transcriptional noise, potentially influenced by mechanical and calcium signals, is an active mechanism facilitating a flexible and robust tissue-wide defense response by allowing individual cells to sample different transcriptional states before committing to specialized functions required to contain the pathogen.

## Methods

### Plant materials and *Sclerotinia sclerotiorum* infection

*Arabidopsis thaliana* Columbia-0 genotype was grown in Jiffy pots under controlled conditions at 22 °C with a 9-h light period at an intensity of 120 μmol/m^2^/s. Leaves of five-week-old plants were inoculated with *S. sclerotiorum* strain 1980 (ATCC18683) using 0.5-cm-wide plugs of Potato Dextrose Agar medium colonized by the fungus. Control samples were prepared identically but with uncolonized agar plugs. The plugs were placed in the center of the adaxial surface of leaves, and plants were incubated in trays sealed with plastic wrap to maintain 80% relative humidity. Entire leaves were collected individually for nuclei extraction 24 h, with two independent biological replicates per treatment.

### Nuclei isolation

One complete leaf per sample was collected and agar plugs were removed. Leaves were transferred to pre-chilled lysis buffer and chopped with a razor blade, as described in [79]. Immediately after 10µm filtration at 1000 g centrifugation, the nuclei were stained with propidium iodide (PI) and isolated using fluorescence-activated cell sorting (FACS) on a Cytek Aurora Cell Sorter 4L form cytometry at the TRI Genotoul facility (Toulouse, France). Sorting was performed using four lasers (355, 405, 488, and 640 nm) with full spectral emission capture. Signals were unmixed to distinguish PI fluorescence from high autofluorescence. Sorting used a 100 µm nozzle at a pressure of 18 pounds per square inch, isolating ~ 6,000 nuclei per sample (Additional file 1: Fig. S15). To assess the quality of isolated nuclei, an aliquot was stained with DAPI and visualized under microscope. Single-nucleus RNA-seq libraries were then immediately prepared from the isolated nuclei.

### Single-nucleus RNA-seq library preparation and sequencing

snRNA-seq libraries were prepared at the GeT-Santé facility (INSERM Rangueil, France) using the Chromium Next GEM Single Cell 3' Kit v3.1 (10 × Genomics, PN-1000268) and the Chromium Next GEM Chip G Single Cell Kit (10x Genomics, PN-1000120), following the manufacturer’s protocol. Briefly, gel beads-in-emulsion (GEMs) were generated using the Chromium Controller device. Nuclear lysis and reverse transcription (RT) occurred within the GEMs, incorporating unique barcodes and UMIs into the cDNA. After RT, GEMs were broken, and cDNA was purified, amplified, and indexed using the dual index kit TT set A (10x Genomics, PN-1000215). To avoid background noise no external RNA spike-ins were added. Sequencing was conducted at the GeT-Plage facility (INRAE Castanet-Tolosan, France) using an Illumina NovaSeq 6000 instrument on one SP flowcell (v1.5) with 100 cycles across two lanes (Illumina cat. #20028401). The corresponding raw reads data is available at the European nucleotide archive under accession number PRJEB88172 [80].

### Sequence processing and filtering

Raw sequencing reads were processed for each sample separately using alevin-fry v0.7.0 [36] and the simpleaf pipeline (https://simpleaf.readthedocs.io/en/latest/). Read counts were processed using the R/Bioconductor packages scran v1.30.2 [81], and scater v1.30.1 [82]. Empty droplets were predicted and removed using the DropletUtils v1.22 package [83]. To remove genes with abnormal read counts or proportions of reads assigned to organelle genomes, a linear model was fit on the pre-filtered data and cells predicted to be outliers were removed. We then applied a hard filter removing nuclei with fewer than 200 UMIs, more than 5% chloroplast assigned genes, or more than 1% mitochondrial assigned genes. Finally, doublet droplets (i.e. barcodes containing multiple nuclei) were predicted and removed using the scDblFinder v1.16 R package [84].

### snRNA analysis

Each sequenced library was normalized separately using scran normalize counts, which encompasses dispersion estimation, Trimmed Mean of M-values (TMM) scaling, and log transformation. To evaluate cell populations the log transformed counts were summarized to informative axes of variance using PCA. To minimize batch effects from multiple *Arabidopsis* samples the PCs were integrated using Harmony v1.2 [37]. To find additional patterns of variation, we summarized the TMM scaled (but not log transformed) counts using non-negative matrix factorization (NMF) in the R package NNLM (github.com/linxihui/NNLM/tree/master). NMF is less commonly used than PCA in single cell analysis, but several studies have shown that it can recover populations of biologically informative cells more effectively than standard PCA analyses [85, 86]. We selected the number of informative factors by imputing masked count values for each gene and selecting the number of factors minimizing the mean squared error. Because results of NMF can differ with different random seeds, we ran NMF with 100 seeds and took the average of frequent clusters as the consensus NMF values as described by Kotliar et al. [87] (Additional file 2: Tables S3, S4). To integrate our results with two previous *Arabidopsis* single cell studies involving pathogens [5, 7], we used the package SingleR v2.4.1 [88] to identify individual cell similarities to their published clusters and predicted cell types. Additionally, we compared each cell with bulk RNA-seq sample counts (using SingleR) from two previous studies to identify cells with similar expression profiles at infection timepoints or lesion locations [5, 7] (Additional file 2: Table S6).

Clustering was performed using Leiden graph clustering [89] in the igraph package v2.0.2 (https://igraph.org/). Because Leiden clustering is sensitive to random seeds, we constructed consensus clusters from 50 random seeds [90]. Furthermore, to avoid bias from arbitrary selection of the clustering resolution value, and to facilitate hierarchical analysis of the clustering, we performed clustering at a range of modularity resolutions (0.01, 0.02, 0.03, 0.04, 0.05, 0.06, 0.08, 0.1, 0.15, 0.3, 0.5, 0.8) and formed hierarchical consensus clusters as described by van Dongen [91]. Cluster parameters were informally selected by comparing results with NMF factors and SingleR annotations from existing datasets and taking the parameters showing the most consistent and biologically informative results. The R data object for this project is available on Zenodo at 10.5281/zenodo.17086215 [92].

### Cluster-specific markers, functional annotation and trajectory analyses

Markers used for enrichment tests between clusters were identified using the FindAllMarkers function in Seurat v5.0.1 [93]. Cluster-specific genes were identified based on Seurat Wilcoxon rank-sum test with adjusted *p*-value ≤ 1E-05, LFC ≥ 2.0 and expression in ≥ 25% of cluster cells as thresholds, and manually curated based on scran’s scoreMarkers function and top contribution to each CNMF (Additional file 2: Table S5). We evaluated expression specificity based on the average log-counts and proportion of cells in which the gene is expressed in each cluster.

Cell type-specific markers were collected and combined from [94] and [39] (Additional file 2: Table S6), and clusters were annotated manually based on the presence or absence of specific markers, as well as typical cell correlations with published studies and log fold changes collated by scPlantDB [5, 7, 38] (Additional file 2: Table S7). For each cluster we annotated cell types and functions using TopGO v2.54 and Fisher’s exact enrichment tests of GO terms and members of PCMDB and scPlantDB classes, and manual evaluation of the presence of known marker genes. Additionally, we assessed co-enrichment of GO terms and curated marker sets using the SuperExactTest package [95] (Additional file 2: Table S8). For differential gene expression analysis, we compared gene expression in control and inoculated cells in each cluster using Seurats FindMarkers function specifying the group_by argument as “treatment”. C08 and C16 lacking control nuclei, we used gene expression in other clusters as reference to call DEGs in these clusters (Additional file 2: Tables S9, S12). Genes were considered differential for adjusted p-val of pairwise Wilcoxon rank test < 0.01, |average(LFC)|> 0.5, and pct.1 or pct.2 > 0.3. Pseudotime trajectory analyses were performed using the TSCAN v1.40.1 [96] and Slingshot v2.10 R packages [41], and DGE tests along pseudotime axes were conducted using tradeSeq [42] (Additional file 2: Tables S13-S15).

### Three prime tag-sequencing (tagseq)

*Arabidopsis thaliana* Col-0 samples corresponding to the periphery of agar plug or disease lesions were collected at time 0 h (without inoculation), 3, 6, 12, and 24 h after inoculation, following the method described in [3]. Leaves from non-inoculated plants under the same conditions were collected as controls simultaneously. Samples were ground using glass beads (2.5 mm) and RNA was extracted using NucleoSpin RNA PLUS extraction kits (Macherey–Nagel) following the manufacturer’s protocols. The quality of the extracted RNA was assessed using Qubit Fluorimeter and Agilent bioanalyzer nanochips. tagseq cDNA library were generated by amplification of the 3’ of mRNA using Lexogen QuantSeq 3' mRNA-Seq Library Prep Kit followed the manufacturer’s protocols. cDNA quality was controlled by Qubit Fluorimeter and Agilent bioanalyzer nanochips. Sequencing was conducted at the Technische Universität München (Freising, Germany) using the NovaSeq Reagent Kits v1.5, Single-Read 1 × 100 base pair reads sequencing on a NovaSeq 6000 instrument. For each experimental condition (inoculated and non-inoculated), three independent biological samples were processed. These reads were simultaneously aligned in bash environment to the *Arabidopsis* Columbia-0 reference genome Araport11, and the *S. sclerotiorum* strain 1980 version 2 genome [97]. Reads were trimmed using FastP 0.23.4 (qualified_quality_phred = 5, length_required = 20), aligned with STAR 2.7.10b (alignIntronMin = 20, outFilterMultimapNmax = 10, outFilterScoreMin = 5) and counts were calculated with featureCounts from subread 2.0.6. The corresponding raw reads data are available at the European nucleotide archive under accession number PRJEB89842 [98], processed data is provided in Additional File 2: Table S10.

Normalization of read counts per gene by TMM was performed using the edgeR package considering total read count by library and by organism [99]. To explore kinetics of differentially expressed genes, differential expression analysis was conducted using edgeR package v 3.32.1 in R 4.0.4 comparing infected plants versus control condition at 3 h, 6 h, 12 h and 24 h post-treatment. Genes with a Benjamini–Hochberg adjusted *P*-value < 0.01 and |Log2 fold change|> 1 were considered significant for differential expression (Additional file 2: Table S11).

### GO term enrichment and gene expression correlation analyses

GO enrichment analyses in clusters were performed as described above for cluster annotation. For enrichment in genes with high entropy in inoculated nuclei, GO enrichment analysis was performed on genes detected in a minimum of 10 nuclei in both control and inoculated samples, using the BINGO 3.0.5 module in Cytoscape 3.9.1. Over-representation of GO terms was assessed with Hypergeometric tests after Benjamini–Hochberg correction at threshold 0.1 (uncorrected *p*-values reported), compared to GO terms in the 15,236 *A. thaliana* expressed genes detected in a minimum of 10 nuclei. For GO enrichment in genes correlated with entropy, the 10,912 DEGs in either snRNA-seq or tagseq were used a reference set. Goodness of fit tests were performed using the gofTest function from the package EnvStats in R. Gene expression along pseudotime trajectories was estimated using 20 equal size pseudobins. For correlation analyses along trajectory 2, expression in bins spanning pseudotime 0.21 to 1 was compared to the median Shannon entropy values in nuclei belonging the corresponding pseudobins using the cor.test function in R with the Spearman method (Additional file 2: Tables S20-S21).

### Gene and cell noise analyses

To quantify transcriptional noise, we used the normalized Shannon entropy and Fano scores (standard deviation divided by the mean). Normalized Shannon entropy for each gene, cell, or cluster was calculated as $\frac{-{\sum }_{i=1}^{n}p\left({c}_{i}\right)\cdot {\mathrm{log}}_{2}p\left({c}_{i}\right) }{{\mathrm{log}}_{2}n}$ where $n$ is the number of categories (e.g. for cell entropy $n$ could be the number of genes) and $p\left({c}_{i}\right)$ is the probability of the observed expression value. To simplify estimation of probabilities, expression values were binned into 20 evenly spaced intervals over all log transformed expression values, and the probability was calculated as the frequency of observing expression in that interval ($n=20$) (Additional file 2: Tables S16-S18).

#### Calcium spatio-temporal quantification

Leaves of four-week-old Arabidopsis expressing the red genetically encoded calcium indicator R-GECO1 [35] were inoculated with *S. sclerotiorum* expressing the green fluorescent protein (GFP). Each leaf originated from a different plant. The progression of fungal infection was tracked using GFP signals and monitored throughout the experiment by taking images every two minutes with an Axiozoom V16 Macroscope (Zeiss) at 7X magnification (Plan-NeoFluar Z 1.0X 0.25NA objective). Fluorescent signals were recorded with an exposure time of 400 ms exposition time per channel: GFP (500-550nm) and RFP (570-640nm). The spatial resolution of the images was 512 × 512 pixels with a pixel size of 37.143 µm. Preprocessing for clustering involved generating a mask to remove the background and the area of the leaf colonized by the fungal mycelium at 18 hpi. The remaining unmasked pixels were then clustered based on their temporal signal variations using the k-means algorithm. The number of clusters was set to three.

## Supplementary Information

Additional file 1: Fig. S1. An overview of nuclei and reads per nucleus before and after filtering. Fig. S2. Clustering and cell-type assignment of the nuclei at three clustering resolutions. Fig. S3. The hierarchical relationship between cluster levels and the cluster sizes for each sample. Fig. S4. Identified cluster specific markers for each of the three clustering levels. Fig. S5. t-SNE plots showing scores of CNMF factors computed on all nuclei, with each sample plotted separately. Fig. S6. Complete list of abundance and enrichment *p*-values of gene ontologies in single nucleus clusters. Fig. S7. Genes involved in camalexin and indole-glucosinolate biosynthesis and their expression values. Fig. S8. Similar trajectories were obtained by estimating the pseudotime trajectories for all cell types together and for the epidermal and mesophyll subsets separately. Fig. S9. Global correlation for genes significantly regulated along pseudotime trajectories supported a good agreement in the transcriptome reprogramming along pseudotime ~ 0.21 to 1 and Tagseq expression between 0 and 24 h post inoculation. Fig. S10. Relationship between different measurements of within nucleus gene expression variability and sequencing statistics. Fig. S11. Distributions of entropy and number of reads per cluster. Fig. S12. t-SNE plots showing entropy and variance metrics per nucleus for each sample. Fig. S13. Relation between genes differentially expressed and entropy increase upon inoculation. Fig. S14. Size distribution of Ca clusters correlates with single nucleus RNA-seq cluster sizes along pseudotime trajectory 2, supporting a correspondence between pseudotime and distance to the fungus. Fig. S15. Isolation of *Arabidopsis thaliana* nuclei by flow cytometry.

Additional file 2: Table S1. General properties for level 1 single-nucleus RNA-seq clusters. Table S2. Evidence used for the annotation of single-nucleus RNA-seq clusters. Table S3. Annotation of consensus non-negative matrix factorization cell clusters. Table S4. Association of genes with CNMF groups. Table S5. Identification of cluster specific genes. Table S6. Annotation of single-nulceus RNA-seq clusters based on cell similarity annotations with other datasets. Table S7. Marker genes used for the annotation of single-nucleus RNA-seq clusters. Table S8. Gene ontology terms enriched in single nucleus RNA-seq clusters and average expression log fold change between control and inoculated cells. Table S9. Genes with differential expression between the control and treated samples. Table S10. Normalized read counts by gene by Trimmed Mean of M-values for tagseq data. Table S11. Differential expression analysis for inoculated versus control samples for the tagseq data. Table S12. List of cell-type specific and common DEGs with GO enrichment analysis. Table S13. Normalized expression along pseudotime trajectory 1 for differentially expressed genes along this trajectory. Table S14. Normalized expression along pseudotime trajectory 2 for differentially expressed genes along this trajectory. Table S15. Normalized expression along pseudotime trajectory 3 for differentially expressed genes along this trajectory. Table S16. Position along pseudotime trajectories and transcriptional noise metrics for each nucleus. Table S17. Number of expressing cells and transcriptional noise metrics for each gene. Table S18. Gene ontologies enriched among genes showing an increase in entropy upon inoculation. Table S19. List of Arabidopsis thaliana genes associated with defense responses. Table S20. Correlation tests between gene expression patterns and transcriptional noise metrics along pseudotime trajectory 2. Table S21. Gene ontologies enriched among genes with expression significantlycorrelated with entropy.

## Data Availability

Raw reads for snRNA-seq data generated in this work are available at the European nucleotide archive (ENA) under accession number PRJEB88172 [80]. Raw reads for tagseq data generated in this work are available at ENA under accession number PRJEB89842 [98]. The R data object for this project is available on Zenodo at [10.5281/zenodo.17086215](https:/doi.org/10.5281/zenodo.17086215) [92]. Other processed data files are provided as supplementary tables (**Additional file 2: Tables S1-S21**).
