# Supplementary material for: Coordinated single-nucleus responses for quantitative disease resistance involve a calcium-associated switch in transcriptional noise
Source: Genome Biol. 2025 Dec 31;27:14. doi: 10.1186/s13059-025-03906-x (PMC12857083; doi:10.1186/s13059-025-03906-x)
Supplement: Supplementary file 1 — Additional file 1: Fig. S1. An overview of nuclei and reads per nucleus before and after filtering. Fig. S2. Clustering and cell-type assignment of the nuclei at three clustering resolutions. Fig. S3. The hierarchical relationship between cluster levels and the cluster sizes for each sample. Fig. S4. Identified cluster specific markers for each of the three clustering levels. Fig. S5. t-SNE plots showing scores of CNMF factors computed on all nuclei, with each sample plotted separately. Fig. S6. Complete list of abundance and enrichment p-values of gene ontologies in single nucleus clusters. Fig. S7. Genes involved in camalexin and indole-glucosinolate biosynthesis and their expression values. Fig. S8. Similar trajectories were obtained by estimating the pseudotime trajectories for all cell types together and for the epidermal and mesophyll subsets separately. Fig. S9. Global correlation for genes significantly regulated along pseudotime trajectories supported a good agreement in the transcriptome reprogramming along pseudotime ~ 0.21 to 1 and Tagseq expression between 0 and 24 h post inoculation. Fig. S10. Relationship between different measurements of within nucleus gene expression variability and sequencing statistics. Fig. S11. Distributions of entropy and number of reads per cluster. Fig. S12. t-SNE plots showing entropy and variance metrics per nucleus for each sample. Fig. S13. Relation between genes differentially expressed and entropy increase upon inoculation. Fig. S14. Size distribution of Ca clusters correlates with single nucleus RNA-seq cluster sizes along pseudotime trajectory 2, supporting a correspondence between pseudotime and distance to the fungus. Fig. S15. Isolation of Arabidopsis thaliana nuclei by flow cytometry. [file 13059_2025_3906_MOESM1_ESM.pdf]

Additional file 1: Supplementary Figures S1-S15

Coordinated single-nucleus responses for quantitative disease resistance involve a calcium-associated switch in transcriptional noise

Darcy A.B. Jones, Florent Delplace, Mehdi Khafif, Matilda Zaffuto, Tou Cheu Xiong, Adelin Barbacci, Sylvain Raffaele

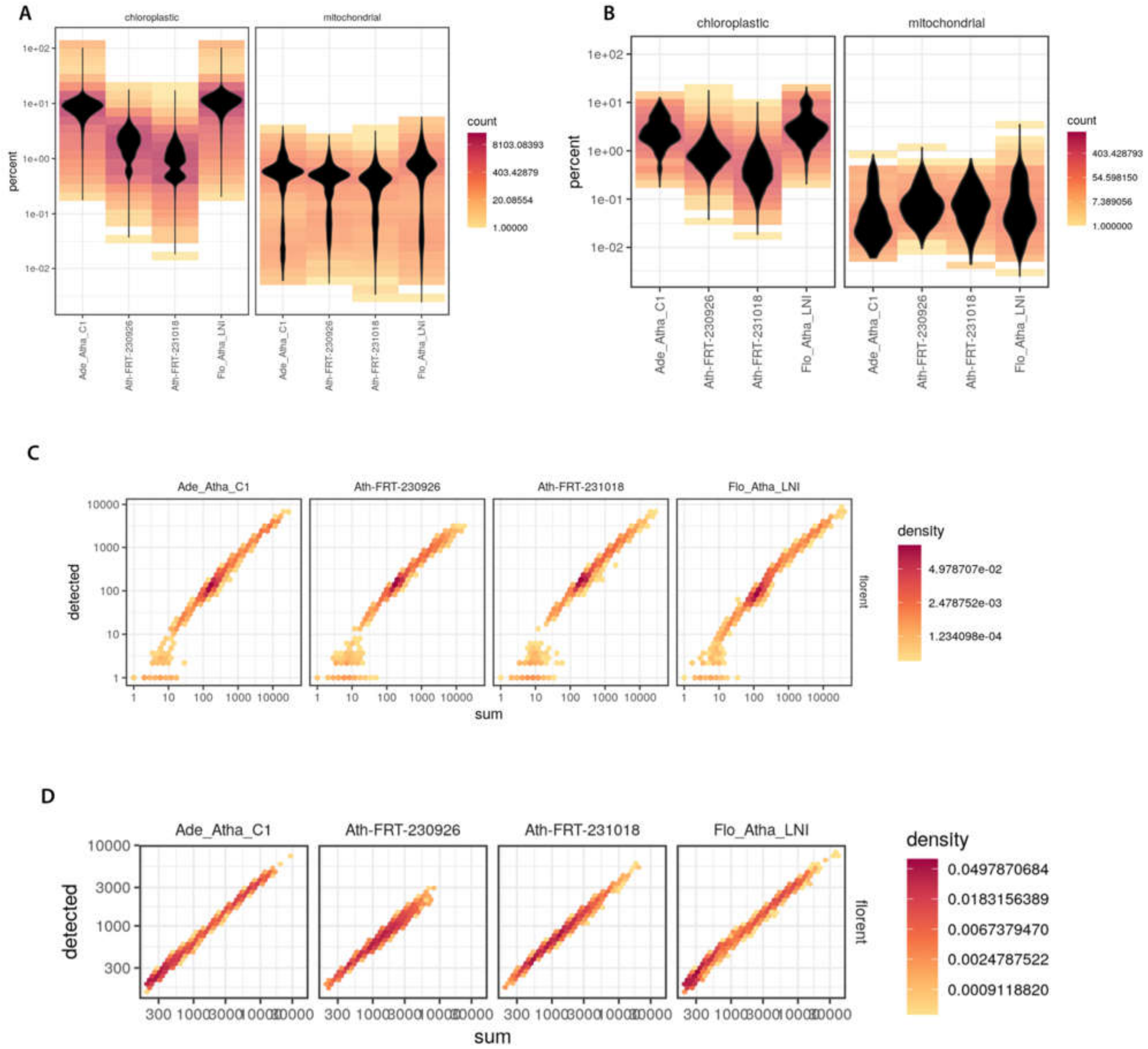

Fig. S1. An overview of nuclei and reads per nucleus before (A, C) and after (B, D) filtering. A and B show the distribution of the percentages of UMIs aligned to the non-nuclear genomes of *A. thaliana.* A higher proportion of chloroplastic reads are observed in the control samples Ade_Atha_C1 and Flo_Atha_LNI (A). this is attenuated after filtering (B). C and D show the relationship between the total number of reads per nucleus (labelled sum), and the number of genes with at least one read supporting it in the nucleus (labelled detected). Ideally this relationship should be linear. C shows a slight non-linearity, and a group of nuclei with very few reads or detected genes. D shows that filtering has effectively removed these potential issues.

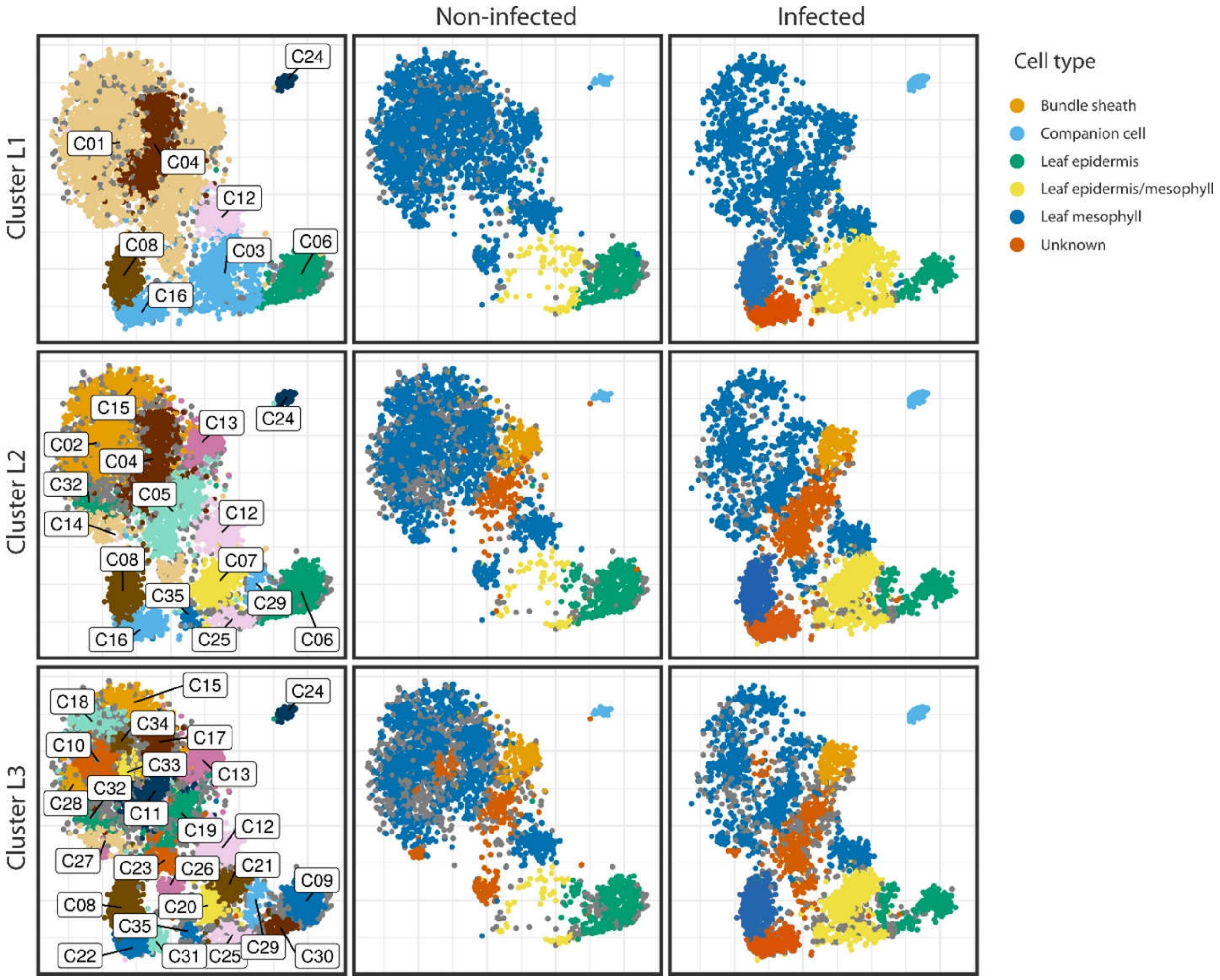

Fig. S2. Clustering and cell-type assignment of the nuclei at three clustering resolutions. The t-SNE scatter plots show nuclei as points with colours indicating either cluster identity (left), or cell type (middle and right). Cluster levels are strictly hierarchical, so that L2 cluster split L1 clusters but do not contain nuclei from multiple L1 clusters. t-SNE condenses high dimensional data (the first 25 HARMONY corrected PCs) into two dimensions so that nuclei with overall similar expression profiles are placed close together. Clusters C08, C16, and C03 (and the equivalent L2 and L3 subclusters) are largely restricted to infected samples (right column), while the infected samples have a lower proportion of C01 cluster member nuclei. Similarly, infected epidermal nuclei n the C06 cluster tend to occupy a restricted region in the t-SNE plot. Note that the colours presented here for clusters and cell-types are different from those in the main text which only display level 1 clustering but are consistent within all supplementary figures.

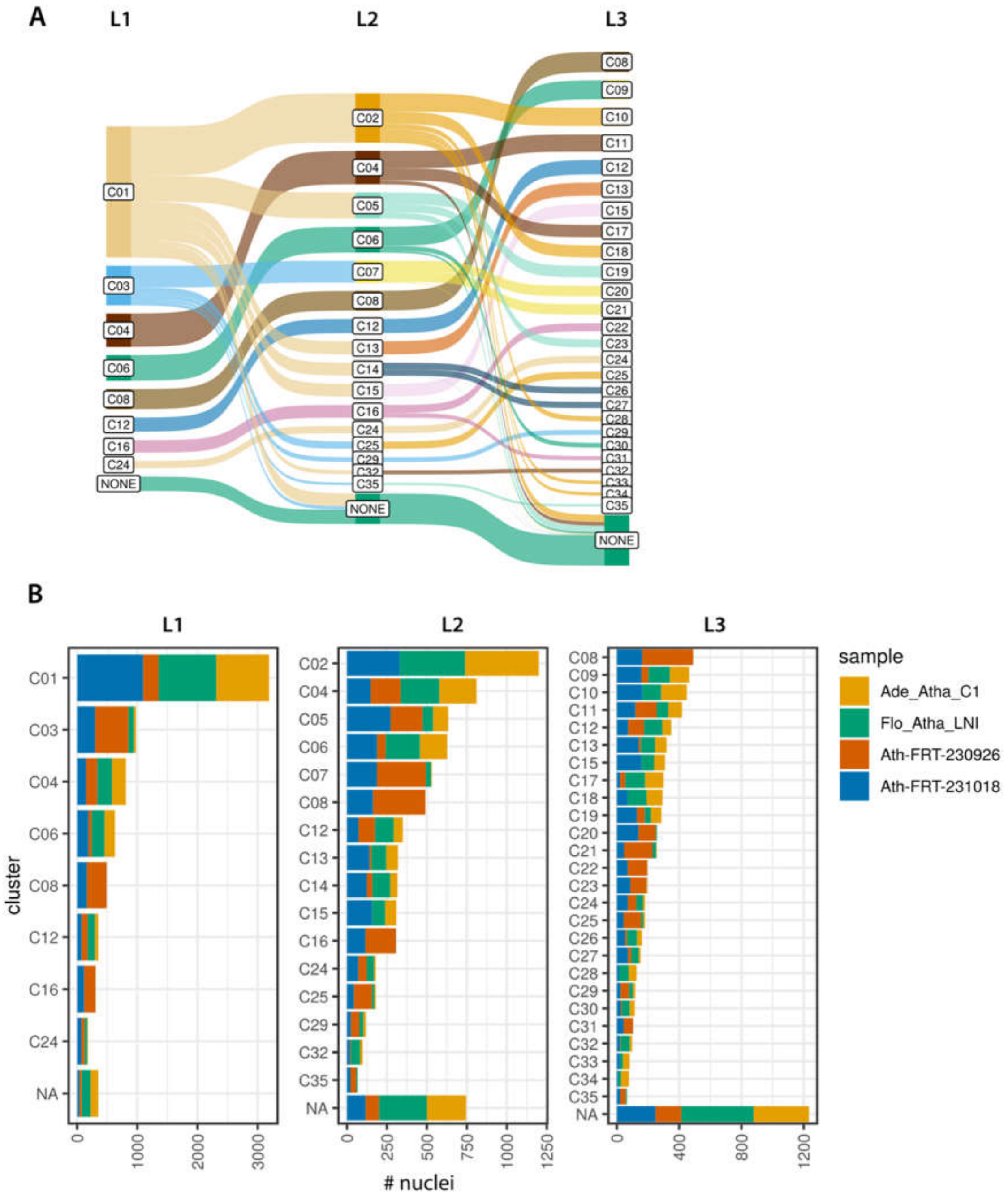

Fig. S3. The hierarchical relationship between cluster levels and the cluster sizes for each sample. (A) Shows how clusters are split at each of the clustering resolutions. Some clusters are relatively stable and persist across all three levels (e.g. C08) while others are split into multiple sub-clusters (e.g. C01, C03). As the clustering resolution increases the proportion of nuclei that cannot be confidently assigned to a single cluster increases (indicated with the "NONE" group). (B) Some clusters have unequal frequencies of nuclei from each sample, but these are more often along the lines of treatment than sample. Note than the infected sample Ath-FRT-230926 is almost missing from C01 and C02. Note also that the non-infected samples tend to have more nuclei that could not be assigned to a cluster (NA/NONE).

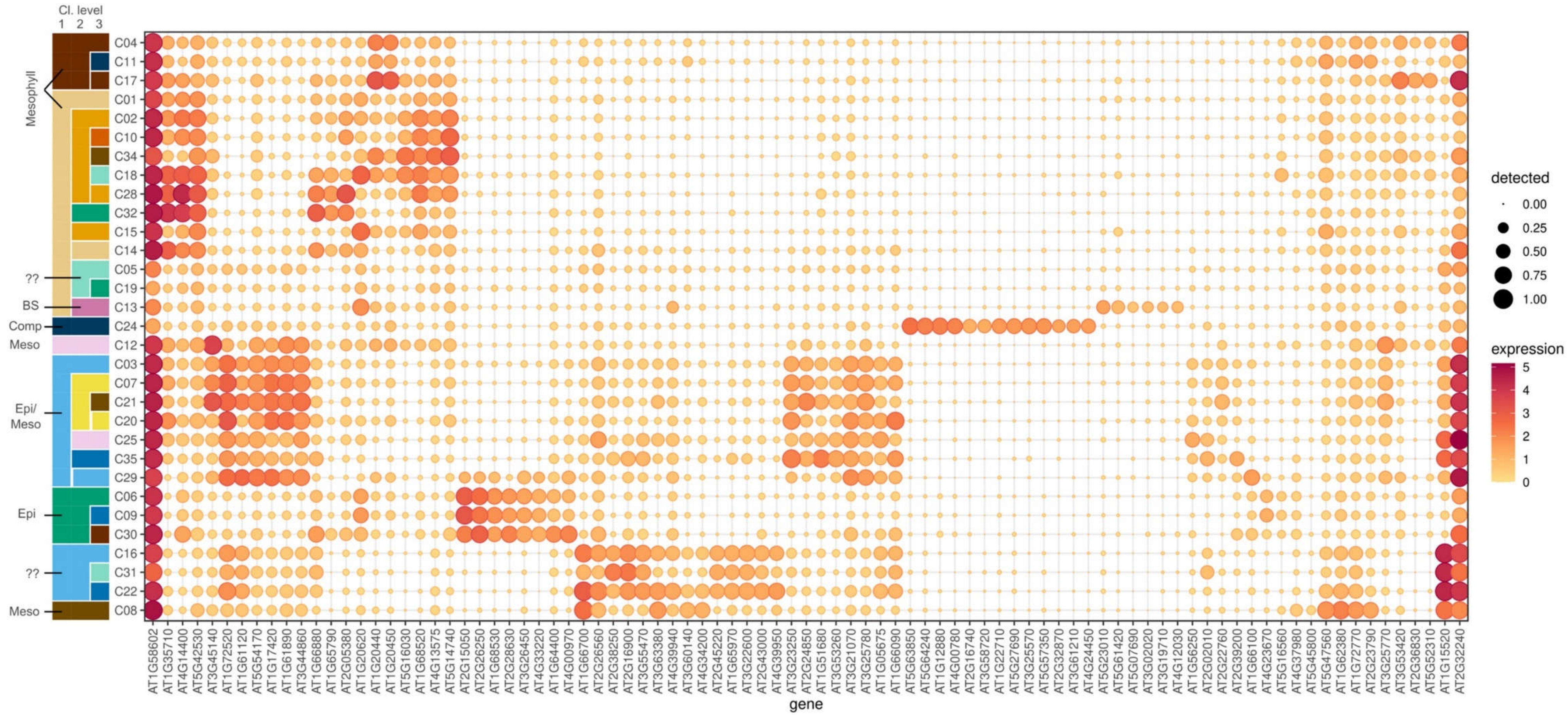

Fig. S4. Identified cluster specific markers for each of the three clustering levels. Circle size indicates the proportion of nuclei within each cluster that the gene was detected at all (at least 1 read pair), while the colour indicates the average normalised log count expression of each gene (including nuclei with 0 reads).

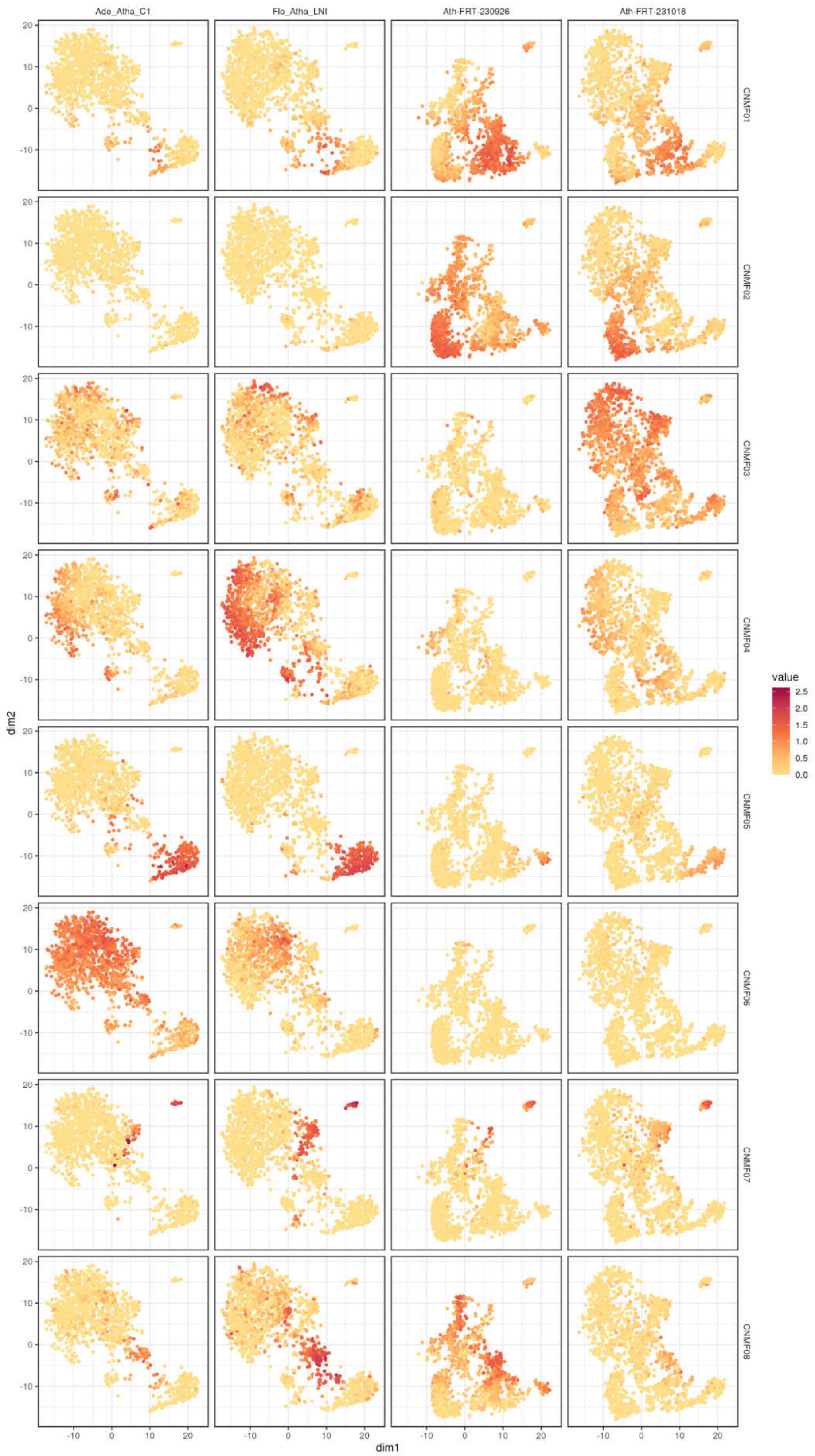

Fig. S5. t-SNE plots showing scores of CNMF factors computed on all nuclei, with each sample plotted separately. Colour indicates which nuclei have high scores for each factor, indicating possible common cellular functions.

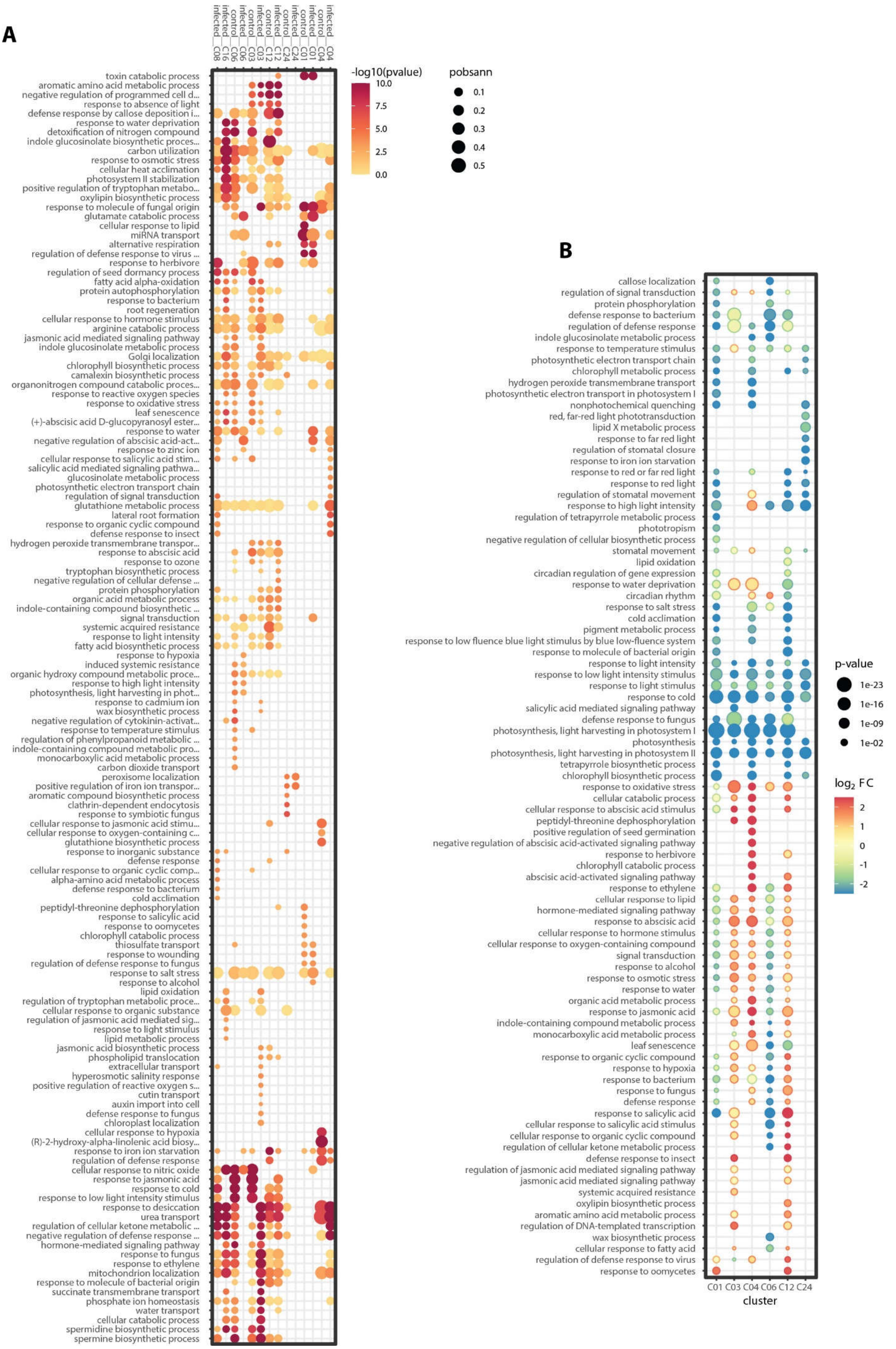
A
infected__C08
infected__C16
control__C06
infected__C06
control__C03
infected__C03
control__C12
infected__C12
control__C24
infected__C24
control__C01
infected__C01
control__C04
infected__C04
toxin catabolic process
aromatic amino acid metabolic process
negative regulation of programmed cell d...
response to absence of light
defense response by callose deposition i...
response to water deprivation
detoxification of nitrogen compound
indole glucosinolate biosynthetic proces...
carbon utilization
response to osmotic stress
cellular heat acclimation
photosystem II stabilization
positive regulation of tryptophan metabo...
oxylipin biosynthetic process
response to molecule of fungal origin
glutamate catabolic process
cellular response to lipid
miRNA transport
alternative respiration
regulation of defense response to virus ...
response to herbivore
regulation of seed dormancy process
fatty acid alpha-oxidation
protein autophosphorylation
response to bacterium
root regeneration
cellular response to hormone stimulus
arginine catabolic process
jasmonic acid mediated signaling pathway
indole glucosinolate metabolic process
Golgi localization
chlorophyll biosynthetic process
camalexin biosynthetic process
organonitrogen compound catabolic proces...
response to reactive oxygen species
response to oxidative stress
leaf senescence
(+)-abscisic acid D-glucopyranosyl ester...
response to water
negative regulation of abscisic acid-act...
response to zinc ion
cellular response to salicylic acid stim...
salicylic acid mediated signaling pathwa...
glucosinolate metabolic process
photosynthetic electron transport chain
regulation of signal transduction
glutathione metabolic process
lateral root formation
response to organic cyclic compound
defense response to insect
hydrogen peroxide transmembrane transpor...
response to abscisic acid
response to ozone
tryptophan biosynthetic process
negative regulation of cellular defense ...
protein phosphorylation
organic acid metabolic process
indole-containing compound biosynthetic ...
signal transduction
systemic acquired resistance
response to light intensity
fatty acid biosynthetic process
response to hypoxia
induced systemic resistance
organic hydroxy compound metabolic proce...
response to high light intensity
photosynthesis, light harvesting in phot...
response to cadmium ion
wax biosynthetic process
negative regulation of cytokinin-activat...
response to temperature stimulus
regulation of phenylpropanoid metabolic ...
indole-containing compound metabolic pro...
monocarboxylic acid metabolic process
carbon dioxide transport
peroxisome localization
positive regulation of iron ion transpor...
aromatic compound biosynthetic process
clathrin-dependent endocytosis
response to symbiotic fungus
cellular response to jasmonic acid stimu...
cellular response to oxygen-containing c...
glutathione biosynthetic process
response to inorganic substance
defense response
cellular response to organic cyclic comp...
alpha-amino acid metabolic process
defense response to bacterium
cold acclimation
peptidyl-threonine dephosphorylation
response to salicylic acid
response to oomycetes
chlorophyll catabolic process
thiosulfate transport
response to wounding
regulation of defense response to fungus
response to salt stress
response to alcohol
lipid oxidation
regulation of tryptophan metabolic proce...
cellular response to organic substance
regulation of jasmonic acid mediated sig...
response to light stimulus
lipid metabolic process
jasmonic acid biosynthetic process
phospholipid translocation
extracellular transport
hyperosmotic salinity response
positive regulation of reactive oxygen s...
cutin transport
auxin import into cell
defense response to fungus
chloroplast localization
cellular response to hypoxia
(R)-2-hydroxy-alpha-linolenic acid biosy...
response to iron ion starvation
regulation of defense response
cellular response to nitric oxide
response to jasmonic acid
response to cold
response to low light intensity stimulus
response to desiccation
urea transport
regulation of cellular ketone metabolic ...
negative regulation of defense response ...
hormone-mediated signaling pathway
response to fungus
response to ethylene
mitochondrion localization
response to molecule of bacterial origin
succinate transmembrane transport
phosphate ion homeostasis
water transport
cellular catabolic process
spermidine biosynthetic process
spermine biosynthetic process
-log10(pvalue)
10.0
7.5
5.0
2.5
0.0
pobsann
0.1
0.2
0.3
0.4
0.5
B
callose localization
regulation of signal transduction
protein phosphorylation
defense response to bacterium
regulation of defense response
indole glucosinolate metabolic process
response to temperature stimulus
photosynthetic electron transport chain
chlorophyll metabolic process
hydrogen peroxide transmembrane transport
photosynthetic electron transport in photosystem I
nonphotochemical quenching
red, far-red light phototransduction
lipid X metabolic process
response to far red light
regulation of stomatal closure
response to iron ion starvation
response to red or far red light
response to red light
regulation of stomatal movement
response to high light intensity
regulation of tetrapyrrole metabolic process
phototropism
negative regulation of cellular biosynthetic process
stomatal movement
lipid oxidation
circadian regulation of gene expression
response to water deprivation
circadian rhythm
response to salt stress
cold acclimation
pigment metabolic process
response to low fluence blue light stimulus by blue low-fluence system
response to molecule of bacterial origin
response to light intensity
response to low light intensity stimulus
response to light stimulus
response to cold
salicylic acid mediated signaling pathway
defense response to fungus
photosynthesis, light harvesting in photosystem I
photosynthesis
photosynthesis, light harvesting in photosystem II
tetrapyrrole biosynthetic process
chlorophyll biosynthetic process
response to oxidative stress
cellular catabolic process
cellular response to abscisic acid stimulus
peptidyl-threonine dephosphorylation
positive regulation of seed germination
negative regulation of abscisic acid-activated signaling pathway
response to herbivore
chlorophyll catabolic process
abscisic acid-activated signaling pathway
response to ethylene
cellular response to lipid
hormone-mediated signaling pathway
response to abscisic acid
cellular response to hormone stimulus
cellular response to oxygen-containing compound
signal transduction
response to alcohol
response to osmotic stress
response to water
organic acid metabolic process
response to jasmonic acid
indole-containing compound metabolic process
monocarboxylic acid metabolic process
leaf senescence
response to organic cyclic compound
response to hypoxia
response to bacterium
response to fungus
defense response
response to salicylic acid
cellular response to salicylic acid stimulus
cellular response to organic cyclic compound
regulation of cellular ketone metabolic process
defense response to insect
regulation of jasmonic acid mediated signaling pathway
jasmonic acid mediated signaling pathway
systemic acquired resistance
oxylipin biosynthetic process
aromatic amino acid metabolic process
regulation of DNA-templated transcription
wax biosynthetic process
cellular response to fatty acid
regulation of defense response to virus
response to oomycetes
C01 C03 C04 C06 C12 C24
cluster
p-value
1e-23
1e-16
1e-09
1e-02
log2 FC
2
1
0
-1
-2

Fig. S6. (A) Complete list of abundance and enrichment p-values of gene ontologies (GO) in single nucleus clusters. Circle sizes indicate the number of genes upregulated in inoculated nuclei harboring selected enriched GO in each cluster, with colors indicating the enrichment p-value from a Fisher exact test. (B) complete list of Differential expression analysis of GO terms in control versus inoculated nuclei. The size of circles indicates differential expression p-value and colors show log2 fold change (FC) of expression. Note that clusters C08 and C16 contain nearly exclusively inoculated nuclei and could therefore not be included in this analysis.

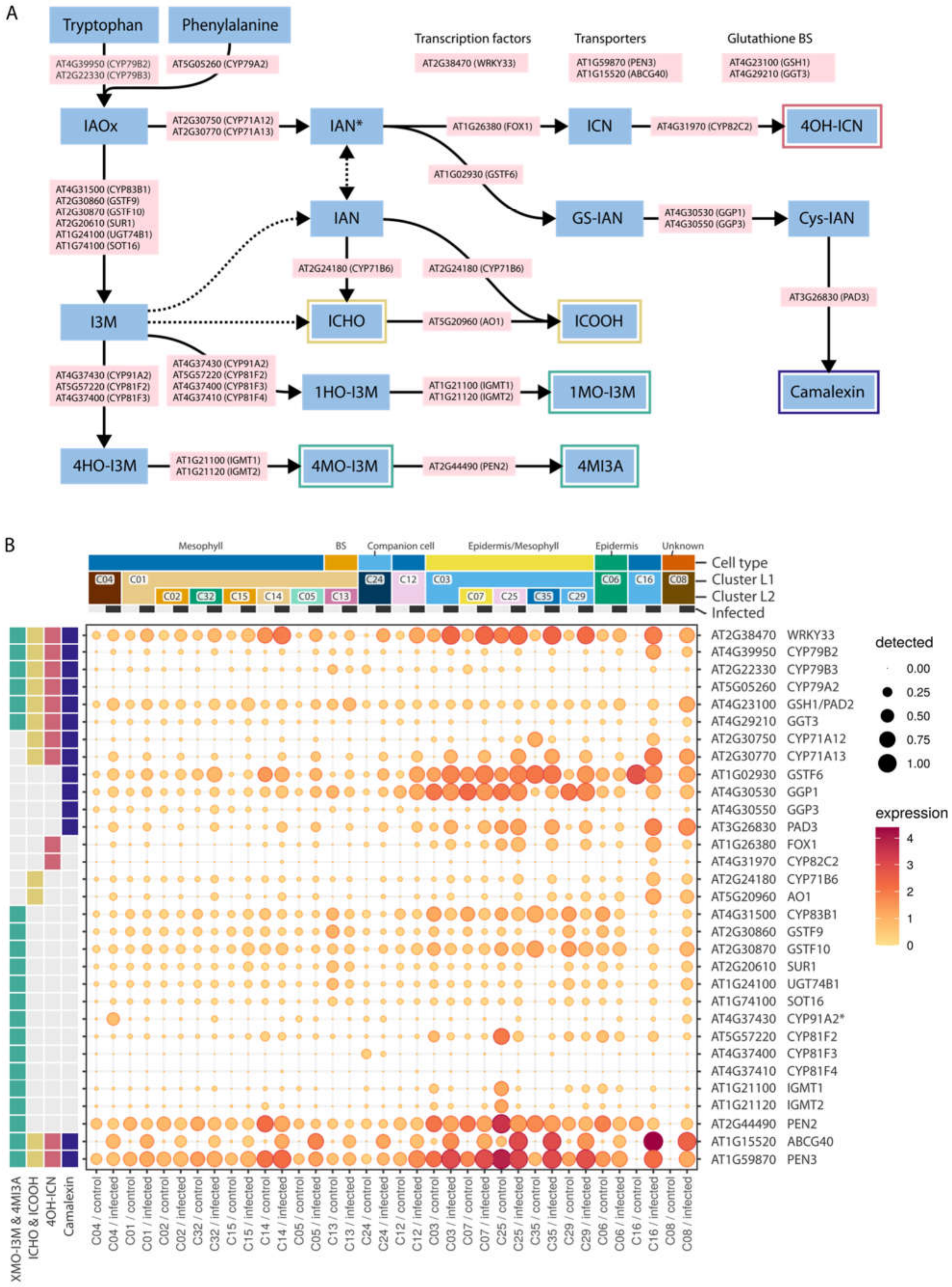

Fig. S7. Genes involved in camalexin and indole-glucosinolate biosynthesis and their expression values. (A) shows a metabolic network of the major genes involved in biosynthesis of seven bioactive indole glucosinolates (highlighted with coloured boxes). Note that for all boxes linking substrates that list more than one gene, the

genes are redundant (E.g. for IAOx → IAN CYP71A12 and CYP71A13 are redundant). The one exception is the link IAOx → I3M, in which only GSTF9 and GSTF10 are redundant, and the genes are otherwise a chain of pathway steps. IAN* indicates one of many possible intermediate IAN forms, but generally thought to be indole cyanohydrin. (B) Shows expression (log transformed normalised counts) of each of these genes in level 1 and 2 clusters, separating the infected and non-infected treatments. Point sizes indicate the proportion of nuclei that the gene was detected in (i.e. the number of read pairs > 0). Note that some clusters contain very few nuclei from the control treatments (particularly C03, C08 and C16 and their subclusters), and point size must be interpreted cautiously in these cases. The colour indicates average normalised log counts (expression) for each gene, including nuclei with 0 read pairs. The boxes on the left margin indicate which lineage of the indole-compounds each gene contributes towards.

Abbreviations:

- **IAOx**, indo-3-acetaldoxime
- **IAN**, indole-3-acetonitrile
- **IAN***, indole-3-cyanohydrin (putative transient intermediate)
- **ICN**, indole carbonyl nitrile
- **4-OH-ICN**, 4-hydroxy-indole-3-carbonylnitrile
- **GS-IAN**, glutathione-indole-3-cyanohydrin
- **Cys-IAN**, indole-3-acetonitrile-cysteine conjugate
- **I3M**, indole-3-yl-methyl glucosinolate (sometimes abbreviated as I3G or IMG)
- **ICHO**, indole-3-carbaldehyde
- **ICOOH**, indole-3-carboxylic acid
- **4HO-I3M**, 4-hydroxy-indole-3-ylmethyl glucosinolate
- **1HO-I3M**, 1-hydroxy-indole-3-ylmethyl-glucosinolate
- **1MO-I3M**, 1-methoxy-indole-3-ylmethyl-glucosinolate (sometimes abbreviated as 1MI3G)
- **4MO-I3M**, 4-methoxy-indole-3-ylmethyl-glucosinolate (sometimes abbreviated as 4MI3G)
- **4MI3A**, 4-methoxyindol-3-ylmethylamine

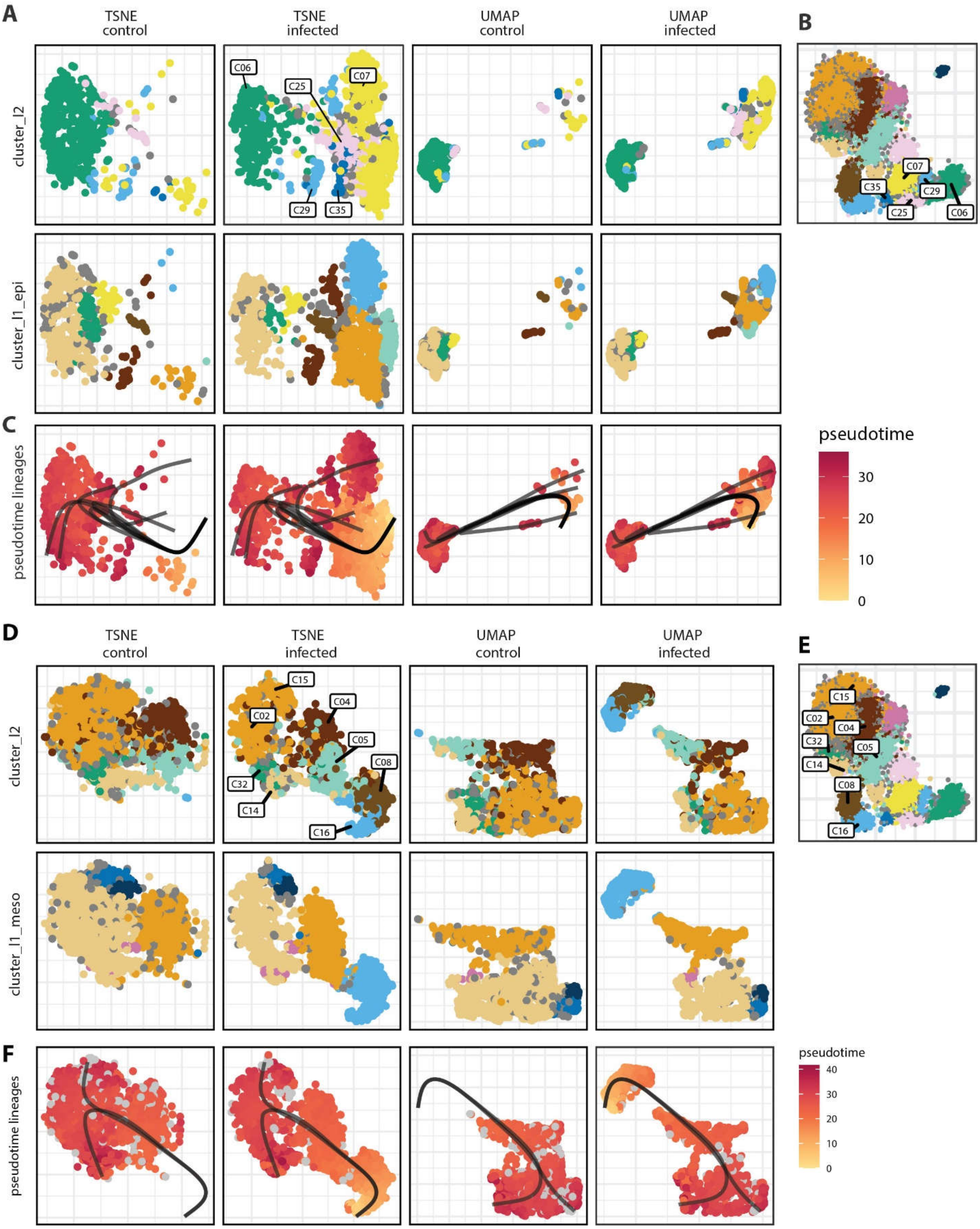

Fig. S8. Similar trajectories were obtained by estimating the pseudotime trajectories for all cell types together and for the epidermal (A-C) and mesophyll (D-F) subsets separately.

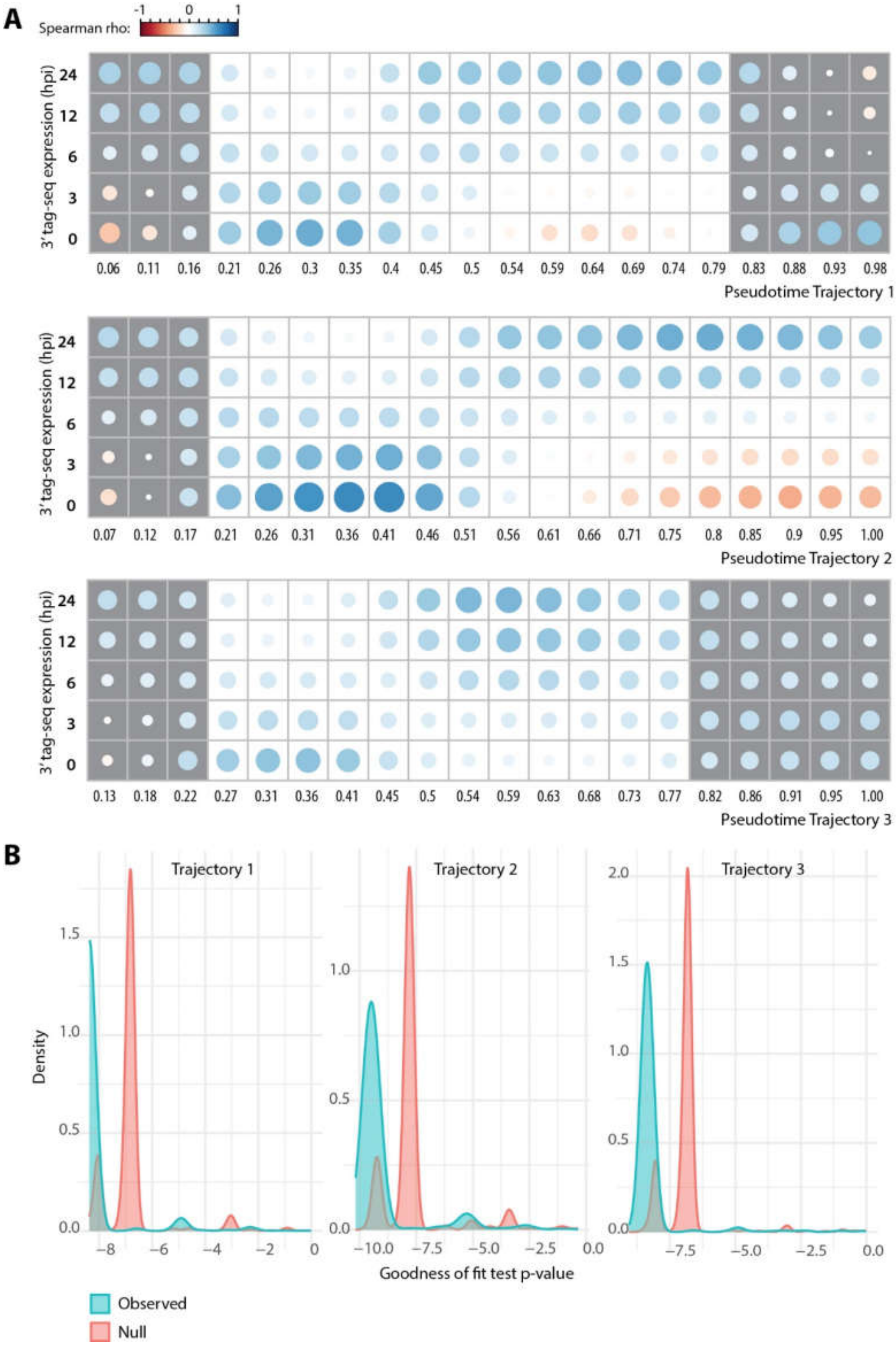

Fig. S9. Global correlation for genes significantly regulated along pseudotime trajectories supported a good agreement in the transcriptome reprogramming along pseudotime ~0.21 to 1 and Tagseq expression between 0 and 24 hours post inoculation (hpi). (A) Correlation plots showing spearman rho as circle size and color for Tagseq timepoints and snRNA-seq pseudotime points. Sections on a grey background showed low fit and were excluded when fitting pseudotime to experimental time course. (B) Distribution of p-values for Goodness of fit tests between Tagseq and pseudotime series. Distribution for observed values and for a null distribution with time points shuffled 10 000 times are shown.

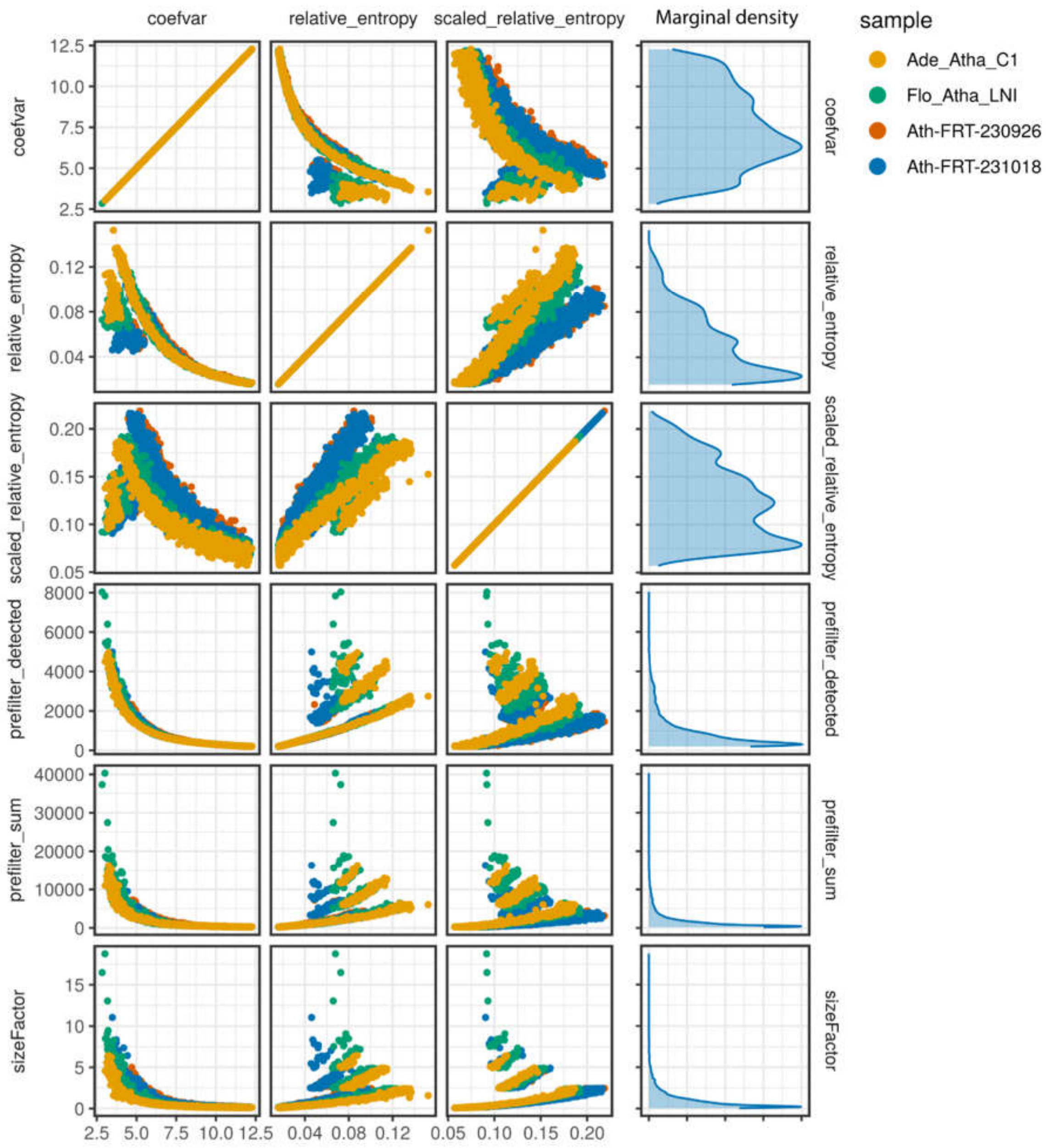

Fig. S10: Relationship between different measurements of within nucleus gene expression variability and sequencing statistics. All variability statistics were calculated from log normalised counts. The coefficient of variation (coefvar, stddev / mean) measures the overall variability across all genes within a single nucleus, it is normally used to compare a single gene across multiple nuclei. Relative entropy indicates the variability of different expression values across all genes, normalised to the maximum possible entropy so that it falls strictly between 0 and 1. A cell with low relative entropy has only a few genes expressed at similar levels, so the complexity of possible cellular function is probably low. In contrast a nucleus with high relative entropy has a more diverse range of expression values (so likely more genes expressed as well) and indicates a more functionally diverse/complex group of cellular activities. Because entropy necessarily is necessarily dependent on the number of genes expressed and the read count, we scale the relative entropy by the sum of log normalised counts per nucleus. "prefilter_detected" indicates the number of genes in each nucleus with expression > 0, "prefilter_sum" indicates the total number of read pairs per nucleus, and "sizeFactor" shows the normalisation factor from TMM to construct normalised counts.

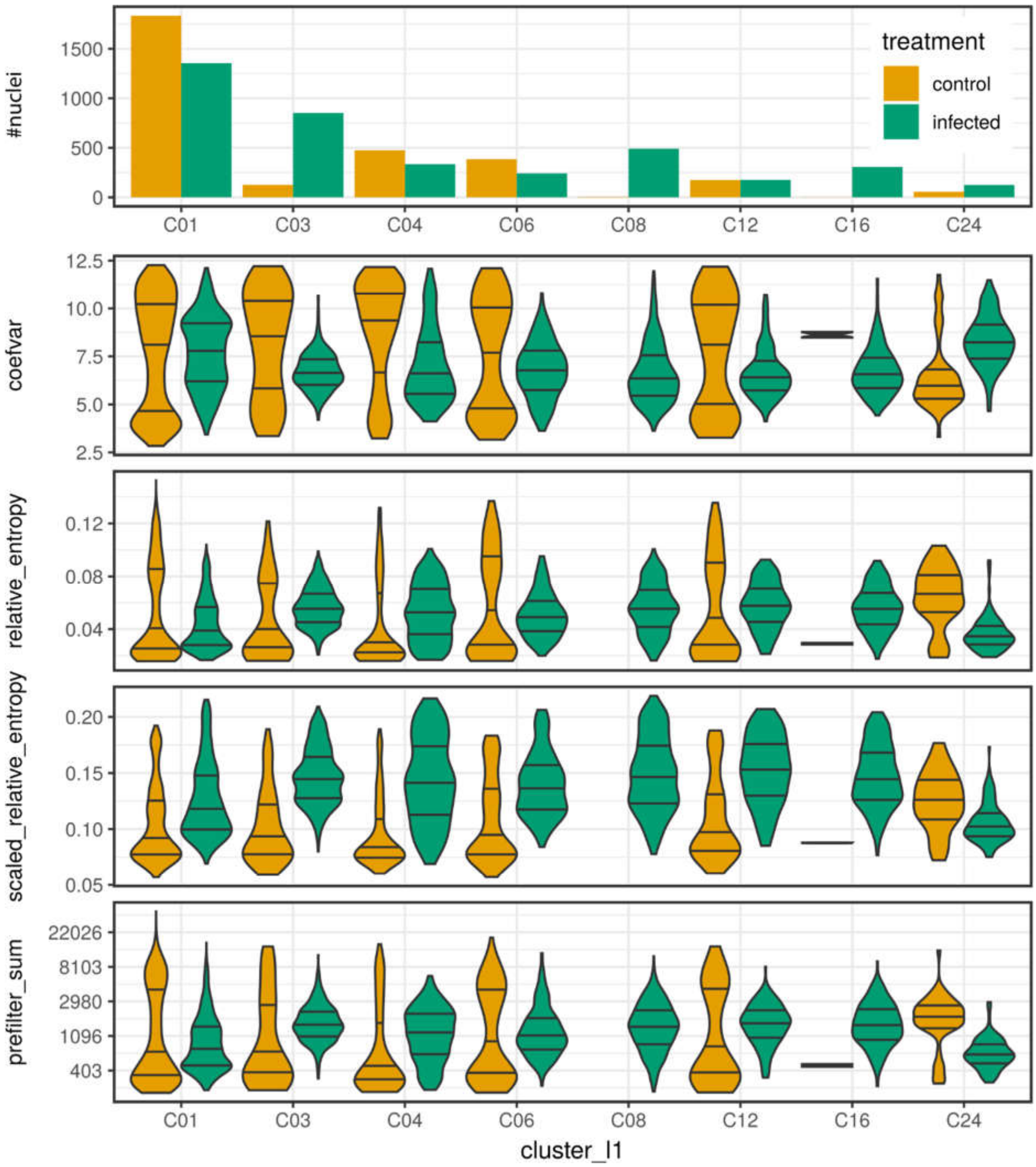

Fig. S11. Distributions of entropy and number of reads per cluster. The coefficient of variation (coefvar) is the within nucleus standard deviation / mean of gene expression. Prefilter sum indicates the total number of read pairs for each nucleus before removing very lowly expressed genes or any normalisation. Scaled relative entropy is the relative entropy divided by the sum of the normalised read counts for each nucleus. Note that C08 has no nuclei from the non-infected samples.

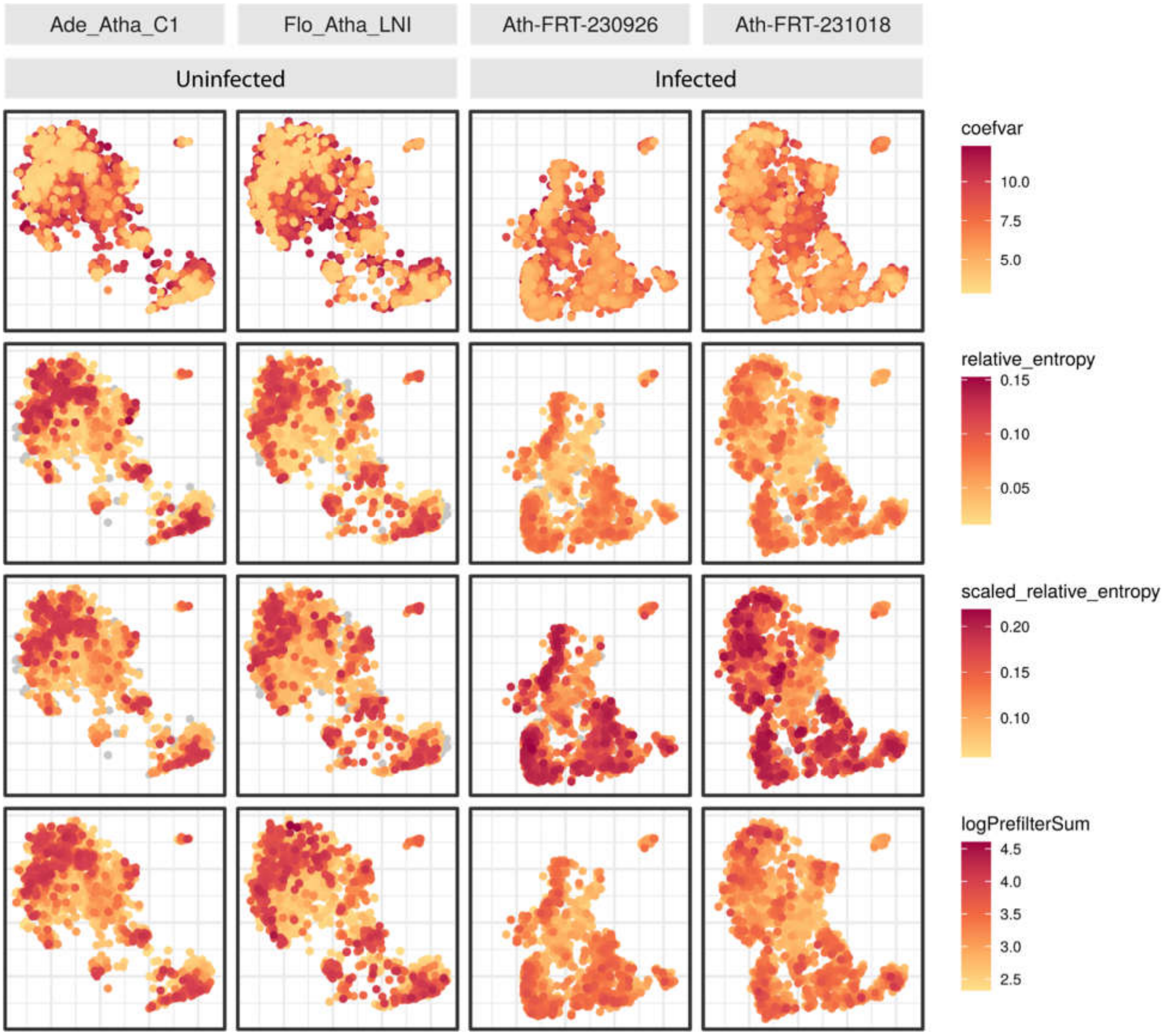

Fig. S12. t-SNE plots showing entropy and variance metrics per nucleus for each sample. The points are ordered so that overplotting does not obscure the subset of nuclei showing high entropy, low co-efficient of variation, or read counts. The coefficient of variation (coefvar) is the within nucleus standard deviation / mean of gene expression. Log prefilter sum indicates the total number of read pairs for each nucleus before removing very lowly expressed genes or any normalisation. Scaled relative entropy is the relative entropy divided by the sum of the normalised read counts for each nucleus.

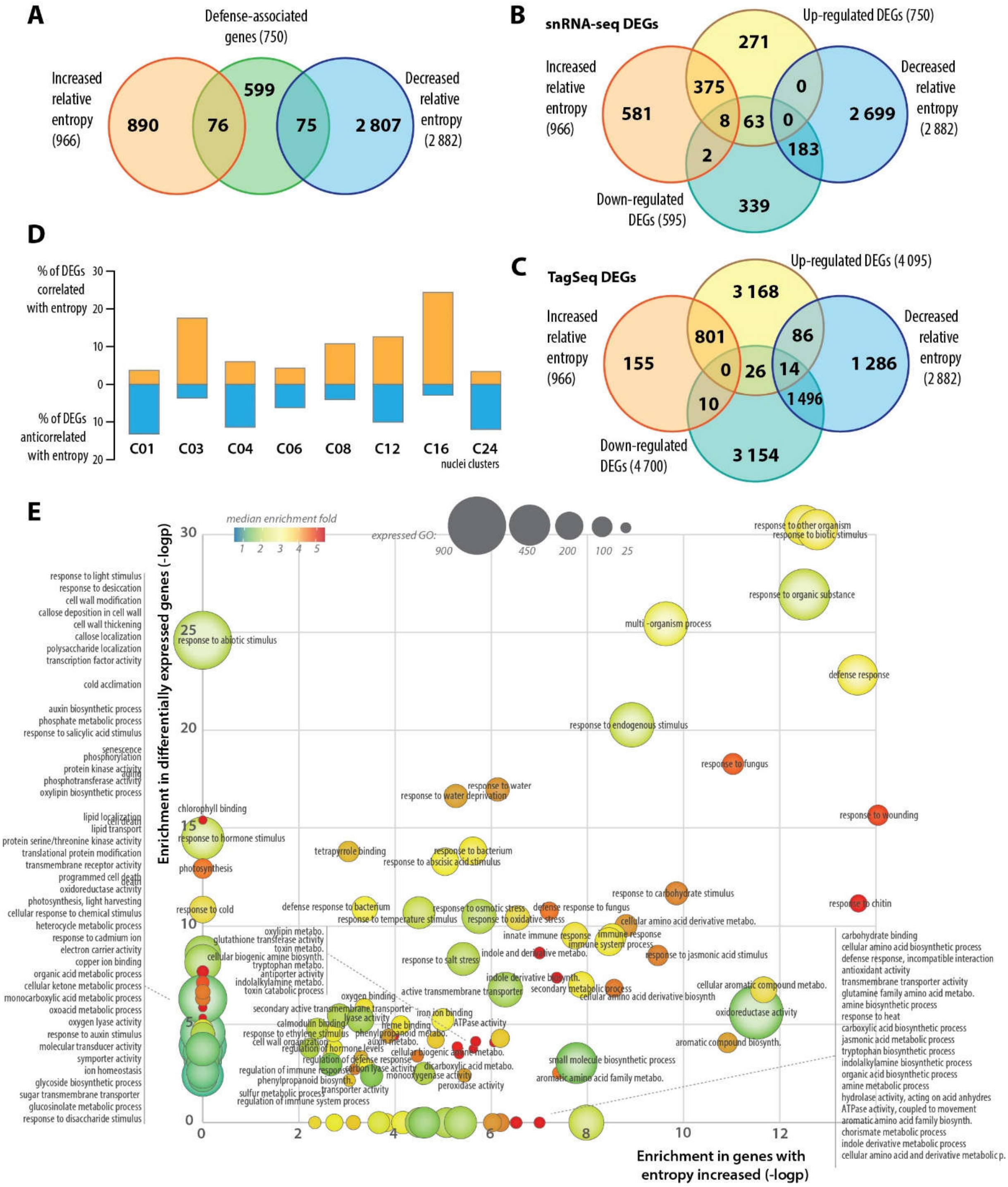

Fig. S13. Relation between genes differentially expressed (DEGs) and entropy increase upon inoculation. (A) Distribution of a set of 750 *A. thaliana* defense-associated genes (Table S19) among genes with increase and decrease of entropy upon inoculation. (B,C) Distribution of DEGs identified by single nucleus RNA-seq (B) and TagSeq (C) among genes with increase and decrease of entropy upon inoculation. (D) Distribution of genes with expression correlated and anticorrelated with entropy among DEGs identified in each nucleus cluster. (E) Gene ontologies significantly enriched among DEGs (Y-axis) and among genes with entropy increase (X-axis).

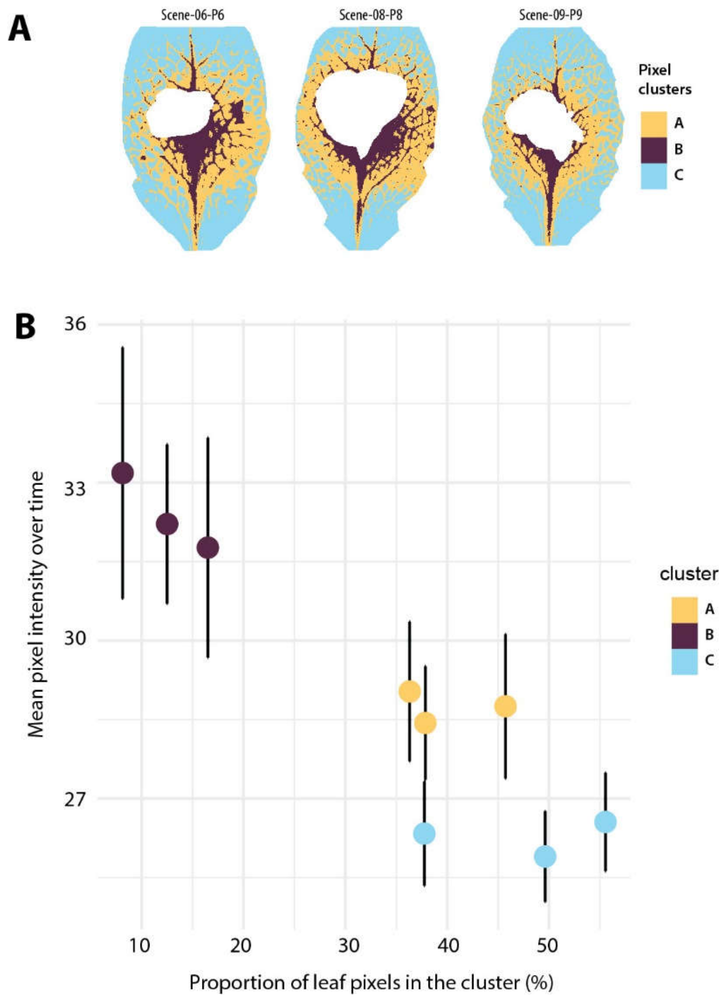

Fig. S14. Size distribution of Ca clusters correlates with single nucleus RNA-seq cluster sizes along pseudotime trajectory 2, supporting a correspondence between pseudotime and distance to the fungus. (A) Clusters of pixels resulting from the integration of R-GECO1 fluorescence over a 20 hours time-course following *S. sclerotiorum* inoculation on three representative leaves. The central white area corresponds to dead cells at the end of imaging. (B) Size of pixel clusters extracted from R-GECO1 time courses in % of the leaf for the three replicates shown in A. Error bars show show standard error from the mean (central dot).

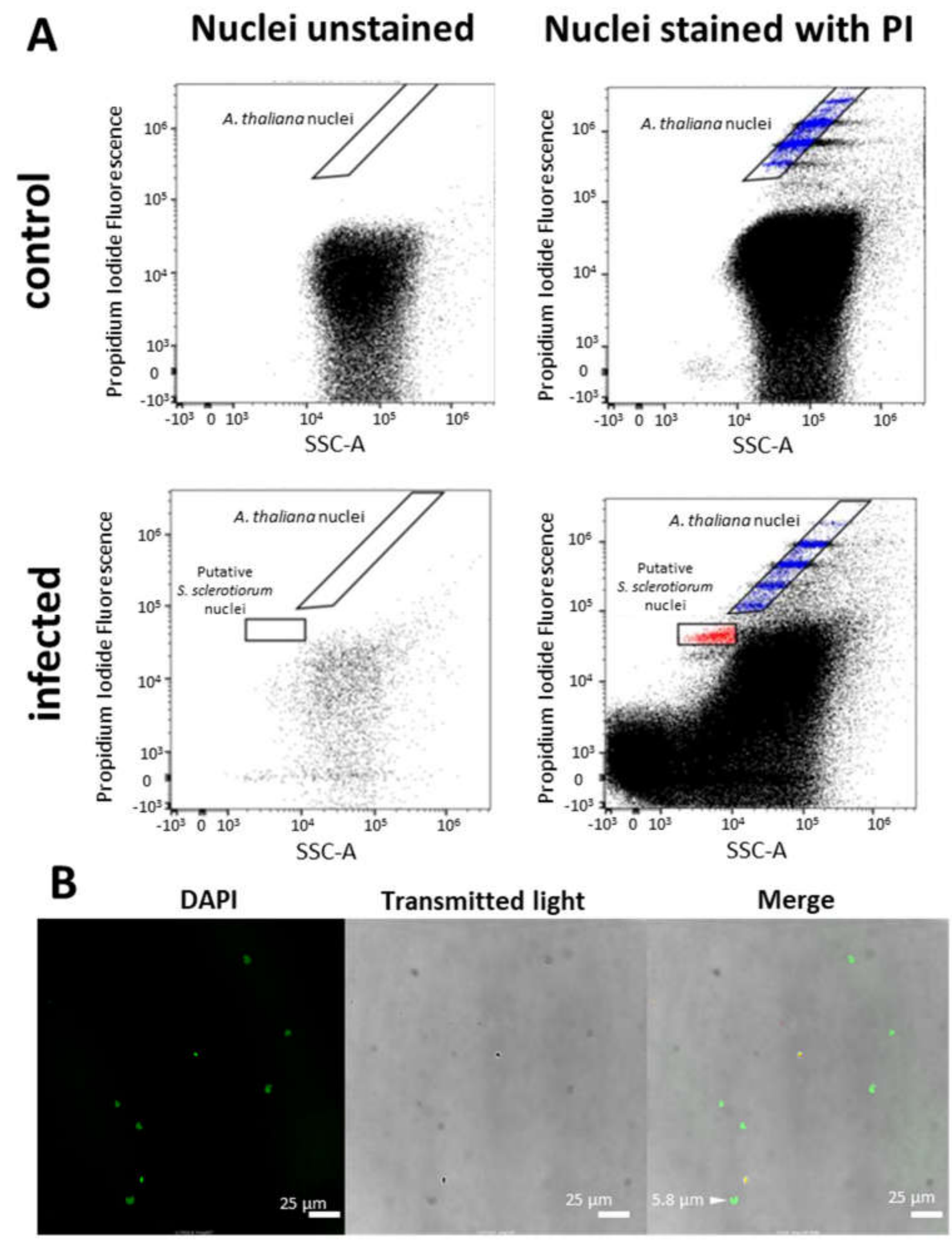

Fig. S15. Isolation of *Arabidopsis thaliana* nuclei by flow cytometry. (A) FACS profiles showing samples from uninfected *A. thaliana* leaves (unstained and PI-stained) and from *S. sclerotiorum*-infected leaves (unstained and PI-stained). The x-axis represents Side Scatter-Area (SSC-A), which reflects granularity and serves here as a proxy for nuclear size. The y-axis displays PI fluorescence intensity, corresponding to DNA content. Gated regions (black outlines) indicate isolated nuclear populations: *A. thaliana* nuclei and putative *S. sclerotiorum* nuclei. Gates were defined based on characteristic granularity and PI signal levels and were used to select nuclei for downstream single-nucleus RNA sequencing (snRNA-seq). Each dot represents a cell object. (B) *A. thaliana* nuclei isolated by FACS and stained with 4′,6-diamidino-2-phenylindole (DAPI), visualized under a light microscope using a 25× objective. The white scale bar represents 25 µm.
